# A second-level diagonal preconditioner for single-step SNPBLUP

**DOI:** 10.1186/s12711-019-0472-8

**Published:** 2019-06-25

**Authors:** Jeremie Vandenplas, Mario P. L. Calus, Herwin Eding, Cornelis Vuik

**Affiliations:** 1 Animal Breeding and Genomics, Wageningen UR, P.O. 338, 6700 AH Wageningen, The Netherlands; 2 CRV BV, Wassenaarweg, 20, 6843 NW Arnhem, The Netherlands; 3 DIAM, TU Delft, Van Mourik Broekmanweg, 6, 2628 XE Delft, The Netherlands

## Abstract

**Background:**

The preconditioned conjugate gradient (PCG) method is an iterative solver of linear equations systems commonly used in animal breeding. However, the PCG method has been shown to encounter convergence issues when applied to single-step single nucleotide polymorphism BLUP (ssSNPBLUP) models. Recently, we proposed a deflated PCG (DPCG) method for solving ssSNPBLUP efficiently. The DPCG method introduces a second-level preconditioner that annihilates the effect of the largest unfavourable eigenvalues of the ssSNPBLUP preconditioned coefficient matrix on the convergence of the iterative solver. While it solves the convergence issues of ssSNPBLUP, the DPCG method requires substantial additional computations, in comparison to the PCG method. Accordingly, the aim of this study was to develop a second-level preconditioner that decreases the largest eigenvalues of the ssSNPBLUP preconditioned coefficient matrix at a lower cost than the DPCG method, in addition to comparing its performance to the (D)PCG methods applied to two different ssSNPBLUP models.

**Results:**

Based on the properties of the ssSNPBLUP preconditioned coefficient matrix, we proposed a second-level diagonal preconditioner that decreases the largest eigenvalues of the ssSNPBLUP preconditioned coefficient matrix under some conditions. This proposed second-level preconditioner is easy to implement in current software and does not result in additional computing costs as it can be combined with the commonly used (block-)diagonal preconditioner. Tested on two different datasets and with two different ssSNPBLUP models, the second-level diagonal preconditioner led to a decrease of the largest eigenvalues and the condition number of the preconditioned coefficient matrices. It resulted in an improvement of the convergence pattern of the iterative solver. For the largest dataset, the convergence of the PCG method with the proposed second-level diagonal preconditioner was slower than the DPCG method, but it performed better than the DPCG method in terms of total computing time.

**Conclusions:**

The proposed second-level diagonal preconditioner can improve the convergence of the (D)PCG methods applied to two ssSNPBLUP models. Based on our results, the PCG method combined with the proposed second-level diagonal preconditioner seems to be more efficient than the DPCG method in solving ssSNPBLUP. However, the optimal combination of ssSNPBLUP and solver will most likely be situation-dependent.

**Electronic supplementary material:**

The online version of this article (10.1186/s12711-019-0472-8) contains supplementary material, which is available to authorized users.

## Background

Since its introduction in the late 1990s [1], the preconditioned conjugate gradient (PCG) method has been the method of choice to solve breeding value estimation models in animal breeding. Likewise, the systems of linear equations of the different single-step single nucleotide polymorphism BLUP (ssSNPBLUP) models are usually solved with the PCG method with a diagonal (also called Jacobi) or block-diagonal preconditioner [2–4]. Several studies [3–6] observed that the PCG method with such a preconditioner applied to ssSNPBLUP is associated with slower convergence. By investigating the reasons for these convergence issues, Vandenplas et al. [4] observed that the largest eigenvalues of the preconditioned coefficient matrix of ssSNPBLUP proposed by Mantysaari and Stranden [7], hereafter referred to as ssSNPBLUP_MS, resulted from the presence of the equations for single nucleotide polymorphism (SNP) effects. In their study, applying a deflated PCG (DPCG) method to ssSNPBLUP_MS solved the convergence issues [4]. In comparison to the PCG method, the DPCG method introduces a second-level preconditioner that annihilates the effect of the largest eigenvalues of the preconditioned coefficient matrix of ssSNPBLUP_MS on the convergence of the iterative solver. After deflation, the largest eigenvalues of the ssSNPBLUP_MS preconditioned deflated coefficient matrix were reduced and close to those of single-step genomic BLUP (ssGBLUP). As a result the associated convergence patterns of ssSNPBLUP were, at least, similar to those of ssGBLUP [4].

While it solves the convergence issues associated with ssSNPBLUP, the DPCG method requires the computation and storage of the so-called Galerkin matrix, which is a dense matrix that could be computationally expensive for very large evaluations and that requires some effort to be implemented in existing software. In addition, as implemented in Vandenplas et al. [4], each iteration of the DPCG method requires two multiplications of the coefficient matrix by a vector, instead of one multiplication for the PCG method. As a result, computing time per iteration with the DPCG method is roughly twice as long as with the PCG method. Accordingly, it is of interest to develop a second-level preconditioner that would reduce the largest eigenvalues of the preconditioned coefficient matrix of ssSNPBLUP at a lower cost than the DPCG method. As such, the aim of this study was to develop a second-level preconditioner that would decrease the unfavourable largest eigenvalues of the preconditioned coefficient matrix of ssSNPBLUP and to compare its performance to the DPCG method. The performance of the proposed second-level preconditioner was tested for two different ssSNPBLUP models.

## Methods

### Data

The two datasets used in this study, hereafter referred to as the reduced and field datasets, were provided by CRV BV (The Netherlands) and are the same as in Vandenplas et al. [4], in which these two datasets are described in detail.

Briefly, for the reduced dataset, the data file included 61,592 ovum pick-up sessions from 4109 animals and the pedigree included 37,021 animals. The 50K SNP genotypes of 6169 animals without phenotypes were available. A total of 9994 segregating SNPs with a minor allele frequency higher than or equal to 0.01 were randomly sampled from the 50K SNP genotypes. The number of SNPs was limited to 9994 to facilitate the computation and the analysis of the left-hand side of the mixed model equations. The univariate mixed model included random effects (additive genetic, permanent environmental and residual), fixed co-variables (heterosis and recombination) and fixed cross-classified effects (herd-year, year-month, parity, age in months, technician, assistant, interval, gestation, session and protocol) [8].

For the field dataset, the data file included 3,882,772 records with a single record per animal. The pedigree included 6,130,519 animals. The genotypes, including 37,995 segregating SNPs, of 15,205 animals without phenotypes and of 75,758 animals with phenotypes were available. The four-trait mixed model included random effects (additive genetic and residual), fixed co-variables (heterosis and recombination) and fixed cross-classified effects (herd x year x season at classification, age at classification, lactation stage at classification, milk yield and month of calving) [9, 10].

### Single-step SNPBLUP models

In this study, we investigated two ssSNPBLUP linear equations systems. The first system was proposed by Mantysaari and Stranden [7] (ssSNPBLUP_MS). This system was also investigated in Vandenplas et al. [4]. The standard multivariate model associated with the ssSNPBLUP_MS system of equations can be written as:

$$\begin{aligned} {\mathbf{y}}={\mathbf{X}}\varvec{\upbeta }+ \left[ \begin{array}{ccc} {\mathbf{W}}_{n}&{}{\mathbf{0}}&{}{\mathbf{0}}\\ {\mathbf{0}}&{}{\mathbf{W}}_{g}&{}{\mathbf{W}}_{g}{\mathbf{M}}_{z} \end{array} \right] \left[ \begin{array}{c} {\mathbf{u}}_{n}\\ {\mathbf{a}}_{g}\\ {\mathbf{g}} \end{array} \right] +{\mathbf{e}} \end{aligned}$$

where the subscripts *g* and *n* refer to genotyped and non-genotyped animals, respectively, ${\mathbf{y}}$ is the vector of records, $\varvec{\upbeta }$ is the vector of fixed effects, ${\mathbf{u}}_{n}$ is the vector of additive genetic effects for non-genotyped animals, ${\mathbf{a}}_{g}$ is the vector of residual polygenic effects for genotyped animals, ${\mathbf{g}}$ is the vector of SNP effects and ${\mathbf{e}}$ is the vector of residuals. The matrices ${\mathbf{X}}$, ${\mathbf{W}}_{n}$ and ${\mathbf{W}}_{g}$ are incidence matrices relating records to their corresponding effects. The matrix ${\mathbf{M}}_{z}$ is equal to ${\mathbf{M}}_{z}={\mathbf{I}}_{t}\otimes {\mathbf{Z}}$, with ${\mathbf{I}}_{t}$ being an identity matrix with size equal to the number of traits $\textit{t}$ and the matrix ${\mathbf{Z}}$ containing the SNP genotypes (coded as 0 for one homozygous genotype, 1 for the heterozygous genotype, or 2 for the alternate homozygous genotype) centred by their observed means.

The system of linear equations for multivariate ssSNPBLUP_MS can be written as follows:

$${\mathbf{C}}_{MS}{\mathbf{x}}_{MS}={\mathbf{b}}_{MS}$$ where

$${\mathbf{C}}_{MS}= \left[ \begin{array}{cccc} {\mathbf{X}}^{\prime }{\mathbf{R}}^{-1}{\mathbf{X}} &{} {\mathbf{X}}^{\prime }_{n}{\mathbf{R}}^{-1}_{n}{\mathbf{W}}_{n} &{} {\mathbf{X}}^{\prime }_{g}{\mathbf{R}}^{-1}_{g}{\mathbf{W}}_{g} &{} {\mathbf{X}}^{\prime }_{g}{\mathbf{R}}^{-1}_{g}{\mathbf{W}}_{g}{\mathbf{M}}_{z}\\ {\mathbf{W}}^{\prime }_{n}{\mathbf{R}}^{-1}_{n}{\mathbf{X}}_{n} &{} {\mathbf{W}}^{\prime }_{n}{\mathbf{R}}^{-1}_{n}{\mathbf{W}}_{n}+\varvec{\Sigma }_{MS}^{11} &{} \varvec{\Sigma }_{MS}^{12}&{} \varvec{\Sigma }_{MS}^{13}\\ {\mathbf{W}}^{\prime }_{g}{\mathbf{R}}^{-1}_{g}{\mathbf{X}}_{g} &{} \varvec{\Sigma }_{MS}^{21} &{} {\mathbf{W}}^{\prime }_{g}{\mathbf{R}}^{-1}_{g}{\mathbf{W}}_{g}+\varvec{\Sigma }_{MS}^{22} &{} {\mathbf{W}}^{\prime }_{g}{\mathbf{R}}^{-1}_{g}{\mathbf{W}}_{g}{\mathbf{M}}_{z}+\varvec{\Sigma }_{MS}^{23} \\ {\mathbf{M}}^{\prime }_{z}{\mathbf{W}}^{\prime }_{g}{\mathbf{R}}^{-1}_{g}{\mathbf{X}}_{g} &{} \varvec{\Sigma }_{MS}^{31} &{} {\mathbf{M}}^{\prime }_{z}{\mathbf{W}}^{\prime }_{g}{\mathbf{R}}^{-1}_{g}{\mathbf{W}}_{g} +\varvec{\Sigma }_{MS}^{32} &{} {\mathbf{M}}^{\prime }_{z}{\mathbf{W}}^{\prime }_{g}{\mathbf{R}}^{-1}_{g}{\mathbf{W}}_{g}{\mathbf{M}}_{z} +\varvec{\Sigma }_{MS}^{33} \end{array} \right]$$

is a symmetric positive (semi-)definite coefficient matrix, $${\mathbf{x}}_{MS}= \left[ \begin{array}{c} \hat{\varvec{\upbeta }}\\ \hat{{\mathbf{u}}}_{n}\\ \hat{{\mathbf{a}}}_{g}\\ \hat{{\mathbf{g}}} \end{array} \right]$$ is the vector of solutions, and

$${\mathbf{b}}_{MS}= \left[ \begin{array}{c} {\mathbf{X}}^{\prime }{\mathbf{R}}^{-1}{\mathbf{y}} \\ {\mathbf{W}}^{\prime }_{n}{\mathbf{R}}^{-1}_{n}{\mathbf{y}}_{n} \\ {\mathbf{W}}^{\prime }_{g}{\mathbf{R}}^{-1}_{g}{\mathbf{y}}_{g} \\ {\mathbf{M}}^{\prime }_{z}{\mathbf{W}}^{\prime }_{g}{\mathbf{R}}^{-1}_{g}{\mathbf{y}}_{g} \end{array} \right]$$

is the right-hand side with

$${\mathbf{R}}^{-1}=\left[ \begin{array}{cc} {\mathbf{R}}^{-1}_{n} &{} {\mathbf{0}}\\ {\mathbf{0}} &{} {\mathbf{R}}^{-1}_{g} \end{array} \right]$$

being the inverse of the residual (co)variance structure matrix. The matrix $\varvec{\Sigma }_{MS}^{-1}$ is the inverse of the covariance matrix associated with ${\mathbf{u}}_{n}$, ${\mathbf{a}}_{g}$ and ${\mathbf{g}}$, and is equal to

$$\begin{aligned}\varvec{\Sigma }_{MS}^{-1}&= \left[ \begin{array}{ccc} \varvec{\Sigma }_{MS}^{11} &{} \varvec{\Sigma }_{MS}^{12} &{} \varvec{\Sigma }_{MS}^{13} \\ \varvec{\Sigma }_{MS}^{21} &{} \varvec{\Sigma }_{MS}^{22} &{} \varvec{\Sigma }_{MS}^{23} \\ \varvec{\Sigma }_{MS}^{31} &{} \varvec{\Sigma }_{MS}^{32} &{} \varvec{\Sigma }_{MS}^{33} \end{array} \right] \\ &= {\mathbf{G}}^{-1}_{0}\otimes \left[ \begin{array}{ccc} {\mathbf{A}}^{nn} &{} {\mathbf{A}}^{ng} &{} {\mathbf{A}}^{ng}{\mathbf{Z}} \\ {\mathbf{A}}^{gn} &{} \frac{1}{w}{\mathbf{A}}^{gg}+\left( 1-\frac{1}{w} \right) {\mathbf{Q}} &{} {{\mathbf{Q}}}{{\mathbf{Z}}} \\ {\mathbf{Z}}^{\prime }{\mathbf{A}}^{gn} &{} {\mathbf{Z}}^{\prime }{\mathbf{Q}} &{} {\mathbf{Z}}^{\prime }{\mathbf{Q}}{\mathbf{Z}}+\frac{m}{1-w}{\mathbf{I}} \end{array} \right]. \end{aligned}$$

The matrix ${\mathbf{Q}}$ is equal to ${\mathbf{Q}}={\mathbf{A}}^{gn} \left( {\mathbf{A}}^{nn} \right) ^{-1}{\mathbf{A}}^{ng}$, with

$${\mathbf{A}}^{-1} = \left[ \begin{array}{cc} {\mathbf{A}}^{nn} &{} {\mathbf{A}}^{ng} \\ {\mathbf{A}}^{gn} &{} {\mathbf{A}}^{gg} \end{array} \right]$$

being the inverse of the pedigree relationship matrix. The parameter *w* is the proportion of variance (due to additive genetic effects) considered as residual polygenic effects and $m=2\sum p_{o}\left( 1-p_{o}\right)$ with $p_{o}$ being the allele frequency of the *o*th SNP.

The second system of linear equations investigated in this study is the system of equations proposed by Gengler et al. [11] and Liu et al. [5], hereafter referred to as ssSNPBLUP_Liu. The system of linear equations for a multivariate ssSNPBLUP_Liu can be written as follows:

$${\mathbf{C}}_{L}{\mathbf{x}}_{L}={\mathbf{b}}_{L}$$

where

$${\mathbf{C}}_{L} = \left[ \begin{array}{cccc} {\mathbf{X}}^{\prime }{\mathbf{R}}^{-1}{\mathbf{X}} &{} {\mathbf{X}}^{\prime }_{n}{\mathbf{R}}^{-1}_{n}{\mathbf{W}}_{n} &{} {\mathbf{X}}^{\prime }_{g}{\mathbf{R}}^{-1}_{g}{\mathbf{W}}_{g} &{} {\mathbf{0}}\\ {\mathbf{W}}^{\prime }_{n}{\mathbf{R}}^{-1}_{n}{\mathbf{X}}_{n} &{} {\mathbf{W}}^{\prime }_{n}{\mathbf{R}}^{-1}_{n}{\mathbf{W}}_{n}+\varvec{\Sigma }^{11}_{L} &{} \varvec{\Sigma }^{12}_{L}&{} \varvec{\Sigma }^{13}_{L}\\ {\mathbf{W}}^{\prime }_{g}{\mathbf{R}}^{-1}_{g}{\mathbf{X}}_{g} &{} \varvec{\Sigma }^{21}_{L} &{} {\mathbf{W}}^{\prime }_{g}{\mathbf{R}}^{-1}_{g}{\mathbf{W}}_{g}+\varvec{\Sigma }^{22}_{L} &{} \varvec{\Sigma }^{23}_{L} \\ {\mathbf{0}} &{} \varvec{\Sigma }^{31}_{L} &{} \varvec{\Sigma }^{32}_{L} &{} \varvec{\Sigma }^{33}_{L} \end{array} \right],$$

$${\mathbf{x}}_{L} = \left[ \begin{array}{c} \hat{\varvec{\upbeta }}\\ \hat{{\mathbf{u}}}_{n}\\ \hat{{\mathbf{u}}}_{g}\\ \hat{{\mathbf{g}}} \end{array} \right],$$

and

$${\mathbf{b}}_{L} = \left[ \begin{array}{c} {\mathbf{X}}^{\prime }{\mathbf{R}}^{-1}{\mathbf{y}} \\ {\mathbf{W}}^{\prime }_{n}{\mathbf{R}}^{-1}_{n}{\mathbf{y}}_{n} \\ {\mathbf{W}}^{\prime }_{g}{\mathbf{R}}^{-1}_{g}{\mathbf{y}}_{g} \\ {\mathbf{0}} \end{array} \right].$$

The matrix $\varvec{\Sigma }^{-1}_{L}$ is equal to

$$\begin{aligned} \varvec{\Sigma }^{-1}_{L} &= \left[ \begin{array}{ccc} \varvec{\Sigma }^{11}_{L} &{} \varvec{\Sigma }^{12}_{L} &{} \varvec{\Sigma }^{13}_{L} \\ \varvec{\Sigma }^{21}_{L} &{} \varvec{\Sigma }^{22}_{L} &{} \varvec{\Sigma }^{23}_{L} \\ \varvec{\Sigma }^{31}_{L} &{} \varvec{\Sigma }^{32}_{L} &{} \varvec{\Sigma }^{33}_{L} \end{array} \right] \\ &= {\mathbf{G}}^{-1}_{0}\otimes \left[ \begin{array}{ccc} {\mathbf{A}}^{nn} &{} {\mathbf{A}}^{ng} &{} {\mathbf{0}}\\ {\mathbf{A}}^{gn} &{} \frac{1}{w}{\mathbf{A}}^{gg}+\left( 1-\frac{1}{w} \right) {\mathbf{Q}} &{} -\frac{1}{w}{\mathbf{A}}^{-1}_{gg}{\mathbf{Z}} \\ {\mathbf{0}} &{} -\frac{1}{w}{\mathbf{Z}}^{\prime }{\mathbf{A}}^{-1}_{gg} &{} \frac{1}{w}{\mathbf{Z}}^{\prime }{\mathbf{A}}^{-1}_{gg}{\mathbf{Z}}+\frac{m}{1-w}{\mathbf{I}} \end{array} \right] \end{aligned}$$

with $${\mathbf{A}}^{-1}_{gg}={\mathbf{A}}^{gg}-{\mathbf{Q}}$$.

It is worth noting that the absorption of the equations associated with ${\hat{\mathbf{g}}}$ of ssSNPBLUP_Liu results in the mixed model equations of single-step genomic BLUP (ssGBLUP) for which the inverse of the genomic relationship matrix is calculated using the Woodbury formula [12]. Several studies (e.g., [13–15]) investigated the possibility of using specific knowledge of a priori variances to weight differently some SNPs in ssGBLUP. Such approaches are difficult to extend to multivariate ssGBLUP, while they can be easily applied in ssSNPBLUP by replacing the matrix ${\mathbf{G}}^{-1}_{0}\otimes \frac{m}{1-w}{\mathbf{I}}$ by a symmetric positive definite matrix ${\mathbf{B}}$ that contains SNP-specific (co)variances obtained by, e.g., Bayesian regression [5].

In the following, matrix ${\mathbf{C}}$ will refer to either ${\mathbf{C}}_{MS}$ or ${\mathbf{C}}_{L}$ (and similarly for the vectors ${\mathbf{x}}$ and ${\mathbf{b}}$). In addition, the matrices ${\mathbf{C}}_{MS}$ and ${\mathbf{C}}_{L}$ have the same structure, and both can be partitioned between the equations associated with SNP effects (*S*) and the equations associated with the other effects (*O*), as follows:

$${\mathbf{C}}= \left[ \begin{array}{cc} {\mathbf{C}}_{OO} &{}{\mathbf{C}}_{OS} \\ {\mathbf{C}}_{SO}&{}{\mathbf{C}}_{SS} \end{array} \right].$$

From this partition, it follows that ${\mathbf{C}}_{MS_{OO}}={\mathbf{C}}_{L_{OO}}$ and that ${\mathbf{C}}_{SO}$, ${\mathbf{C}}_{OS}$, and ${\mathbf{C}}_{SS}$ are dense matrices.

### The PCG method

The PCG method is an iterative method that uses successive approximations to obtain more accurate solutions for a linear system at each iteration step [16]. The preconditioned systems of the linear equations of ssSNPBLUP_MS and of ssSNPBLUP_Liu have the form:

1 $$\begin{aligned} {\mathbf{M}}^{-1}{\mathbf{C}}{\mathbf{x}}={\mathbf{M}}^{-1}{\mathbf{b}}, \end{aligned}$$

where ${\mathbf{M}}$ is a (block-)diagonal preconditioner.

In this study, the (block-)diagonal preconditioner ${\mathbf{M}}$ is defined as:

$$\begin{aligned} {\mathbf{M}}= \left[ \begin{array}{cc} {\mathbf{M}}_{ff} &{} {\mathbf{0}} \\ {\mathbf{0}} &{} {\mathbf{M}}_{rr} \end{array} \right] = \left[ \begin{array}{cc} diag \left( {\mathbf{C}}_{ff} \right) &{} {\mathbf{0}} \\ {\mathbf{0}} &{} block\_diag \left( {\mathbf{C}}_{rr} \right) \end{array} \right] \end{aligned}$$

where the subscripts *f* and *r* refer to the equations associated with fixed and random effects, respectively and $block\_diag\left( {\mathbf{C}}_{rr} \right)$ is a block-diagonal matrix with blocks corresponding to equations for different traits within a level (e.g., an animal).

After *k* iterations of the PCG method applied to the Eq. (1), the error is bounded by [16, 17]:

$$\begin{aligned} \left| \left| {\mathbf{x}} -\hat{{\mathbf{x}}}_{k} \right| \right| _{{\mathbf{C}}} \le 2 \left| \left| {\mathbf{x}} -\hat{{\mathbf{x}}}_{0} \right| \right| _{{\mathbf{C}}} \left( \frac{\sqrt{\kappa \left( {\mathbf{C}}_{M} \right) }-1}{\sqrt{\kappa \left( {\mathbf{C}}_{M} \right) }+1} \right) ^{k} \end{aligned}$$

where ${\mathbf{C}}_{M}={\mathbf{M}}^{-1}{\mathbf{C}}$, $\left| \left| {\mathbf{x}} \right| \right| _{{\mathbf{C}}}$ is the ${\mathbf{C}}$-norm of ${\mathbf{x}}$, defined as $\sqrt{{\mathbf{x}}^{\prime }{\mathbf{C}}{\mathbf{x}}}$, and $\kappa \left( {\mathbf{C}}_{M}\right)$ is the effective spectral condition number of ${\mathbf{C}}_{M}$, that is defined as $\frac{\lambda _{max}\left( {\mathbf{C}}_{M}\right) }{\lambda _{min}\left( {\mathbf{C}}_{M}\right) }$ with $\lambda _{max}\left( {\mathbf{C}}_{M}\right)$ ($\lambda _{min}\left( {\mathbf{C}}_{M}\right)$) being the largest (smallest) non-zero eigenvalue of ${\mathbf{C}}_{M}$.

### The deflated PCG method

Vandenplas et al. [4] showed that the largest eigenvalues of the ssSNPBLUP_MS preconditioned coefficient matrix ${\mathbf{C}}_{M}$ were larger than those of the ssGBLUP preconditioned coefficient matrix, while the smallest eigenvalues were similar. This resulted in larger effective condition numbers $\kappa \left( {\mathbf{C}}_{M}\right)$ and convergence issues for ssSNPBLUP_MS. As applied by Vandenplas et al. [4], the DPCG method annihilates the largest unfavourable eigenvalues of the ssSNPBLUP_MS preconditioned coefficient matrix ${\mathbf{C}}_{M}$, which resulted in effective condition numbers and convergence patterns of ssSNPBLUP_MS similar to those of ssGBLUP solved with the PCG method. The preconditioned deflated linear systems of ssSNPBLUP_MS and of ssSNPBLUP_Liu mixed model equations have the form:

$$\begin{aligned} {\mathbf{M}}^{-1}{\mathbf{P}}{\mathbf{C}}{\mathbf{x}}={\mathbf{M}}^{-1}{\mathbf{P}}{\mathbf{b}}, \end{aligned}$$

where ${\mathbf{P}}$ is a second-level preconditioner, called the deflation matrix, equal to ${\mathbf{P}}={\mathbf{I}}-{\mathbf{C}}{\mathbf{Z}}_{d}{\mathbf{E}}^{-1}{\mathbf{Z}}_{d}^{\prime }$, with the matrix ${\mathbf{Z}}_{d}$ being the deflation-subspace matrix as defined in Vandenplas et al. [4] and ${\mathbf{E}}={\mathbf{Z}}_{d}^{\prime }{\mathbf{C}}{\mathbf{Z}}_{d}$ being the Galerkin matrix.

### A second-level diagonal preconditioner

The DPCG method requires the computation and the storage of the Galerkin matrix ${\mathbf{E}}$, which is computationally expensive for very large evaluations [4]. Furthermore, as implemented in Vandenplas et al. [4], each iteration of the DPCG method requires two multiplications of the coefficient matrix ${\mathbf{C}}$ by a vector, instead of one multiplication for the PCG method. Here, our aim is to develop another second-level preconditioner that decreases the largest eigenvalues of the preconditioned coefficient matrix ${\mathbf{C}}_{M}$ at a lower cost than the DPCG method and results in smaller effective condition numbers and better convergence patterns.

To achieve this aim, we introduce a second-level diagonal preconditioner defined as:

$$\begin{aligned} {\mathbf{D}}= \left[ \begin{array}{cc} k_{O}{\mathbf{I}}_{OO} &{}{\mathbf{0}} \\ {\mathbf{0}}&{}k_{S}{\mathbf{I}}_{SS} \end{array} \right] = k_{O} \left[ \begin{array}{cc} {\mathbf{I}}_{OO} &{}{\mathbf{0}} \\ {\mathbf{0}}&{}\frac{k_{S}}{k_{O}}{\mathbf{I}}_{SS} \end{array} \right] = k_{O} \tilde{{\mathbf{D}}} \end{aligned}$$

where ${\mathbf{I}}_{OO}$ is an identity matrix of size equal to the number of equations that are not associated with SNP effects, ${\mathbf{I}}_{SS}$ is an identity matrix of size equal to the number of equations that are associated with SNP effects, $k_{O}$ and $k_{S}$ are real positive numbers and $$\tilde{{\mathbf{D}}} = \left[ \begin{array}{cc} {\mathbf{I}}_{OO} &{}{\mathbf{0}} \\ {\mathbf{0}}&{}\frac{k_{S}}{k_{O}}{\mathbf{I}}_{SS} \end{array} \right]$$. Possible values for $k_{O}$ and $k_{S}$ are discussed below.

Therefore, the preconditioned system of Eq. (1) is modified as follows:

2 $$\begin{aligned} {\mathbf{D}}^{-1}{\mathbf{M}}^{-1}{\mathbf{C}}{\mathbf{x}}={\mathbf{D}}^{-1}{\mathbf{M}}^{-1}{\mathbf{b}}. \end{aligned}$$

Hereafter, we show that the proposed second-level preconditioner ${\mathbf{D}}$ applied to ssSNPBLUP systems of equations results in smaller effective condition numbers by decreasing the largest eigenvalues of the preconditioned coefficient matrices. For simplicity, the symmetric preconditioned coefficient matrix ${\mathbf{D}}^{-1/2}{\mathbf{M}}^{-1/2}{\mathbf{C}}{\mathbf{M}}^{-1/2}{\mathbf{D}}^{-1/2} = {\mathbf{D}}^{-1/2}\tilde{{\mathbf{C}}}{\mathbf{D}}^{-1/2}$ with $\tilde{{\mathbf{C}}} = {\mathbf{M}}^{-1/2}{\mathbf{C}}{\mathbf{M}}^{-1/2}$ is used instead of ${\mathbf{D}}^{-1}{\mathbf{M}}^{-1}{\mathbf{C}}$. Indeed, these two matrices have the same spectrum, i.e., the same set of eigenvalues. In addition, the effective condition number of ${\mathbf{D}}^{-1/2}\tilde{{\mathbf{C}}}{\mathbf{D}}^{-1/2}$, $\kappa \left( {\mathbf{D}}^{-1/2}\tilde{{\mathbf{C}}}{\mathbf{D}}^{-1/2} \right)$, is equal to the effective condition number of $\tilde{{\mathbf{D}}}^{-1/2}\tilde{{\mathbf{C}}}\tilde{{\mathbf{D}}}^{-1/2}$, $\kappa \left( \tilde{{\mathbf{D}}}^{-1/2}\tilde{{\mathbf{C}}}\tilde{{\mathbf{D}}}^{-1/2} \right)$, because:

$$\begin{aligned} \kappa \left( {\mathbf{D}}^{-1/2}\tilde{{\mathbf{C}}}{\mathbf{D}}^{-1/2} \right)&= {} \frac{\lambda _{max}\left( {\mathbf{D}}^{-1/2}\tilde{{\mathbf{C}}} {\mathbf{D}}^{-1/2}\right) }{\lambda _{min}\left( {\mathbf{D}}^{-1/2}\tilde{{\mathbf{C}}} {\mathbf{D}}^{-1/2}\right) } \\ &= {} \frac{\lambda _{max}\left( k_{O}^{-1} \tilde{{\mathbf{D}}}^{-1/2}\tilde{{\mathbf{C}}} \tilde{{\mathbf{D}}}^{-1/2}\right) }{\lambda _{min}\left( k_{O}^{-1} \tilde{{\mathbf{D}}}^{-1/2}\tilde{{\mathbf{C}}} \tilde{{\mathbf{D}}}^{-1/2}\right) } \\ &= {} \frac{\lambda _{max}\left( \tilde{{\mathbf{D}}}^{-1/2}\tilde{{\mathbf{C}}} \tilde{{\mathbf{D}}}^{-1/2}\right) }{\lambda _{min}\left( \tilde{{\mathbf{D}}}^{-1/2}\tilde{{\mathbf{C}}} \tilde{{\mathbf{D}}}^{-1/2}\right) } \\ &= {} \kappa \left( \tilde{{\mathbf{D}}}^{-1/2}\tilde{{\mathbf{C}}}\tilde{{\mathbf{D}}}^{-1/2} \right) \end{aligned}$$

with $$\lambda _{min}\left( k_{O}^{-1} \tilde{{\mathbf{D}}}^{-1/2}\tilde{{\mathbf{C}}} \tilde{{\mathbf{D}}}^{-1/2}\right) = k_{O}^{-1}\lambda _{min}\left( \tilde{{\mathbf{D}}}^{-1/2}\tilde{{\mathbf{C}}} \tilde{{\mathbf{D}}}^{-1/2}\right)$$, and $$\lambda _{max}\left( k_{O}^{-1} \tilde{{\mathbf{D}}}^{-1/2}\tilde{{\mathbf{C}}} \tilde{{\mathbf{D}}}^{-1/2}\right) = k_{O}^{-1}\lambda _{max}\left( \tilde{{\mathbf{D}}}^{-1/2}\tilde{{\mathbf{C}}} \tilde{{\mathbf{D}}}^{-1/2}\right)$$.

The result is that $\kappa \left( {\mathbf{D}}^{-1/2}\tilde{{\mathbf{C}}}{\mathbf{D}}^{-1/2} \right)$ depends only on $\tilde{{\mathbf{D}}}$ and therefore only on the $k_{O}/k_{S}$ ratio.

Regarding the largest eigenvalues of the preconditioned coefficient matrix ${\mathbf{D}}^{-1/2}\tilde{{\mathbf{C}}}{\mathbf{D}}^{-1/2}$ or equivalently of ${\mathbf{D}}^{-1}{\mathbf{M}}^{-1}{\mathbf{C}}$, the effect of the second-level preconditioner ${\mathbf{D}}$ on $\lambda _{max} \left( {\mathbf{D}}^{-1/2}\tilde{{\mathbf{C}}}{\mathbf{D}}^{-1/2} \right)$ can be analysed using the Gershgorin circle theorem [18]. From this theorem, it follows that the largest eigenvalue of the preconditioned coefficient matrix ${\mathbf{D}}^{-1/2}\tilde{{\mathbf{C}}}{\mathbf{D}}^{-1/2}$ is bounded by, for all *i*th and *j*th equations:

3 $$\begin{aligned} \lambda _{max} \left( {\mathbf{D}}^{-1/2}\tilde{{\mathbf{C}}}{\mathbf{D}}^{-1/2} \right) \le {max }_{i} \left\{ {\mathbf{D}}^{-1/2}_{ii}\tilde{{\mathbf{C}}}_{ii}{\mathbf{D}}^{-1/2}_{ii}+ \sum _{j \ne i } | {\mathbf{D}}^{-1/2}_{ii}\tilde{{\mathbf{C}}}_{ij}{\mathbf{D}}^{-1/2}_{jj} | \right\} . \end{aligned}$$

Partitioned between the equations associated with SNP effects (*S*) and with the other effects (*O*), it follows from Eq. (3) that $\lambda _{max} \left( {\mathbf{D}}^{-1/2}\tilde{{\mathbf{C}}}{\mathbf{D}}^{-1/2} \right)$ has the following lower and upper bounds (see Additional file 1 for the derivation):

4 $$\begin{aligned} k_{O}^{-1} \lambda _{max} \left( \tilde{{\mathbf{C}}}_{OO} \right) \le \lambda _{max} \left( {\mathbf{D}}^{-1/2}\tilde{{\mathbf{C}}}{\mathbf{D}}^{-1/2} \right) \le k_{O}^{-1} max \left\{ a,b \right\} , \end{aligned}$$

with $$a= {max }_{k} \left\{ \tilde{{\mathbf{C}}}_{OO_{kk}}+ \sum _{j \ne k } | \tilde{{\mathbf{C}}}_{OO_{kj}} | + \sqrt{\frac{k_{O}}{k_{S}}}\sum _{j \ne k } | \tilde{{\mathbf{C}}}_{OS_{kj}} | \right\}$$,

$b= max _{1} \left\{ \frac{k_{O}}{k_{S}}\tilde{{\mathbf{C}}}_{SS_{ll}}+ \frac{k_{O}}{k_{S}}\sum _{j \ne l } | \tilde{{\mathbf{C}}}_{SS_{lj}} | + \sqrt{\frac{k_{O}}{k_{S}}}\sum _{j \ne l } | \tilde{{\mathbf{C}}}_{SO_{lj}} | \right\}$ and *k* and *l* referring to the equations not associated with and associated with SNP effects, respectively.

Therefore, for a fixed value of $k_{O}$, the upper bound of $\lambda _{max} \left( {\mathbf{D}}^{-1/2}\tilde{{\mathbf{C}}}{\mathbf{D}}^{-1/2} \right)$ will decrease with decreasing $k_{O}/k_{S}$ ratios, up to the lowest upper bound $k_{O}^{-1} max _{k} \left\{ \tilde{{\mathbf{C}}}_{OO_{kk}}+ \sum _{j \ne k } | \tilde{{\mathbf{C}}}_{OO_{kj}} | \right\}$, that is the upper bound of $k_{O}^{-1}\lambda _{max} \left( \tilde{{\mathbf{C}}}_{OO} \right)$.

Nevertheless, decreasing the largest eigenvalue does not (necessarily) mean decreasing the effective condition number $\kappa \left( {\mathbf{D}}^{-1/2}\tilde{{\mathbf{C}}}{\mathbf{D}}^{-1/2} \right)$, because $\lambda _{min} \left( {\mathbf{D}}^{-1/2}\tilde{{\mathbf{C}}}{\mathbf{D}}^{-1/2} \right)$ could decrease at the same rate as, or faster than $\lambda _{max} \left( {\mathbf{D}}^{-1/2}\tilde{{\mathbf{C}}}{\mathbf{D}}^{-1/2} \right)$ leading to constant or larger $\kappa \left( {\mathbf{D}}^{-1/2}\tilde{{\mathbf{C}}}{\mathbf{D}}^{-1/2} \right)$. As such, it is required that $\lambda _{min} \left( {\mathbf{D}}^{-1/2}\tilde{{\mathbf{C}}}{\mathbf{D}}^{-1/2} \right)$ decreases at a lower rate, remains constant, or even increases, when $\lambda _{max} \left( {\mathbf{D}}^{-1/2}\tilde{{\mathbf{C}}}{\mathbf{D}}^{-1/2} \right)$ decreases with decreasing $k_{O}/k_{S}$ ratios. This would be achieved if $\lambda _{min} \left( {\mathbf{D}}^{-1/2}\tilde{{\mathbf{C}}}{\mathbf{D}}^{-1/2} \right)$ is independent of $k_{S}$. Hereafter, we formulate a sufficient condition to ensure that $\lambda _{min} \left( {\mathbf{D}}^{-1/2}\tilde{{\mathbf{C}}}{\mathbf{D}}^{-1/2} \right) = k_{O}^{-1}\lambda _{min} \left( \tilde{{\mathbf{C}}} \right)$ for any $k_{O}/k_{S}$ ratio.

Let the matrix $\tilde{{\mathbf{V}}}$ be a matrix containing (columnwise) all the eigenvectors of $\tilde{{\mathbf{C}}}$ sorted according to the ascending order of their associated eigenvalues. The set of eigenvalues of $\tilde{{\mathbf{C}}}$ sorted according to their ascending order is hereafter called spectrum of $\tilde{{\mathbf{C}}}$. The matrix $\tilde{{\mathbf{V}}}$ can be partitioned into a matrix $\tilde{{\mathbf{V}}}_{1}$ storing eigenvectors associated with eigenvalues at the left-hand side of the spectrum (that includes the smallest eigenvalues) of $\tilde{{\mathbf{C}}}$ and a matrix $\tilde{{\mathbf{V}}}_{2}$ storing eigenvectors at the right-hand side of the spectrum (that includes the largest eigenvalues)of $\tilde{{\mathbf{C}}}$, and between equations associated with SNP effects or not, as follows:

$$\tilde{{\mathbf{V}}} = \left[ \begin{array}{cc} \tilde{{\mathbf{V}}}_{1}&\tilde{{\mathbf{V}}}_{2} \end{array} \right] = \left[ \begin{array}{cc} \tilde{{\mathbf{V}}}_{O1} &{} \tilde{{\mathbf{V}}}_{O2}\\ \tilde{{\mathbf{V}}}_{S1} &{} \tilde{{\mathbf{V}}}_{S2}\\ \end{array} \right].$$

A sufficient condition to ensure that $\lambda _{min} \left( {\mathbf{D}}^{-1/2}\tilde{{\mathbf{C}}}{\mathbf{D}}^{-1/2} \right) = k_{O}^{-1}\lambda _{min} \left( \tilde{{\mathbf{C}}} \right)$ is that $\tilde{{\mathbf{V}}}_{S1}={\mathbf{0}}$, $\tilde{{\mathbf{V}}}_{O2}={\mathbf{0}}$ and that all eigenvalues associated with an eigenvector of $\tilde{{\mathbf{V}}}_{2}$ are equal to, or larger than $\frac{k_{S}}{k_{O}}\lambda _{min}\left( \tilde{{\mathbf{C}}}\right)$ (see Additional file 2 for proof). Therefore, the effective condition numbers $\kappa \left( {\mathbf{D}}^{-1/2}\tilde{{\mathbf{C}}}{\mathbf{D}}^{-1/2} \right)$ will decrease with decreasing $k_{O}/k_{S}$ ratios until the largest eigenvalue $\lambda _{max} \left( {\mathbf{D}}^{-1/2}\tilde{{\mathbf{C}}}{\mathbf{D}}^{-1/2} \right)$ reaches its lower bound $k_{O}^{-1}\lambda _{max} \left( \tilde{{\mathbf{C}}}_{OO} \right)$, as long as the sufficient condition is satisfied. In practice, the pattern of the matrix $\tilde{{\mathbf{V}}}$ will never be as required by the sufficient condition, because the submatrices $\tilde{{\mathbf{C}}}_{OS}$ and $\tilde{{\mathbf{C}}}_{SO}$ contain non-zero entries. However, this sufficient condition is helpful to formulate the expectation that convergence of the models will improve with decreasing $k_{O}/k_{S}$ ratios up to a point that can either be identified from the analyses or by computing the eigenvalues of $\tilde{{\mathbf{C}}}$.

### Analyses

Eigenvalues and eigenvectors of ssSNPBLUP_MS preconditioned coefficient matrices ${\mathbf{D}}^{-1/2}\tilde{{\mathbf{C}}}{\mathbf{D}}^{-1/2}$ with values of $k_{S}$ from 1 to $10^{5}$ (and $k_{O}=1$) were computed for the reduced dataset using the subroutine *dsyev* provided by Intel(R) Math Kernel Library (MKL) 11.3.2.

Using the matrix-free version of the software developed in Vandenplas et al. [4], the system of ssSNPBLUP_MS and ssSNPBLUP_Liu equations for the reduced and field datasets were solved with the PCG and DPCG methods together with the second-level preconditioner ${\mathbf{D}}$ for different values of $k_{S}$ (with $k_{O}=1$). The second-level preconditioner ${\mathbf{D}}$ was implemented by combining it with the preconditioner ${\mathbf{M}}$, as $\tilde{{\mathbf{M}}}={\mathbf{D}}{\mathbf{M}}$. Accordingly, its implementation has no additional costs for an iteration of the PCG and DPCG methods. The DPCG method was applied with 5 SNP effects per subdomain [4]. To illustrate the effect of $k_{O}$, the system of ssSNPBLUP_MS equations was also solved for the reduced dataset with the PCG method and different values of $k_{O}$ (with $k_{S}=1$). For both the PCG and DPCG methods, the iterative process stopped when the relative residual norm was smaller than $10^{-6}$. For all systems, the smallest and largest eigenvalues that influence the convergence of the iterative methods were estimated using the Lanczos method based on information obtained from the (D)PCG method [16, 19, 20]. Effective condition numbers were computed from these estimates [17].

All real vectors and matrices were stored using double precision real numbers, except for the preconditioner, which was stored using single precision real numbers. All computations were performed on a computer with 528 GB and running RedHat 7.4 (x86_64) with an Intel Xeon E5-2667 (3.20 GHz) processor with 16 cores. The number of OpenMP threads was limited to 5 for both datasets. Time requirements are reported for the field dataset. All reported times are indicative, because they may have been influenced by other jobs running simultaneously on the computer.

## Results

### Reduced dataset

**Fig. 1:**
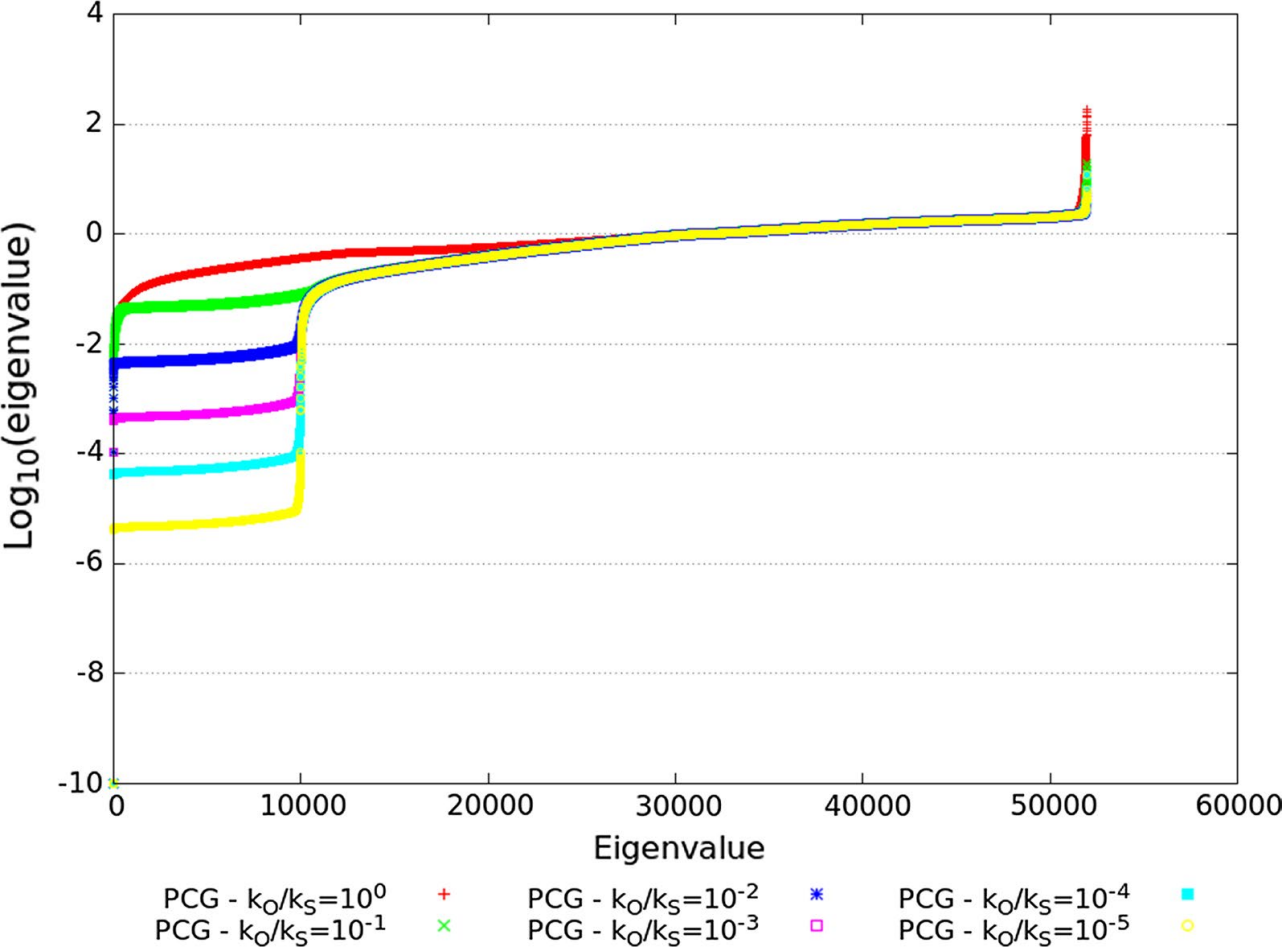
Eigenvalues of different preconditioned coefficient matrices ${\tilde{{\mathrm {C}}}}$ for the reduced dataset. Eigenvalues of the preconditioned coefficient matrices of ssSNPBLUP_MS are depicted on a logarithmic scale. All eigenvalues less than $10^{-10}$ were set to $10^{-10}$. Eigenvalues are sorted in ascending order

The spectra of the ssSNPBLUP_MS preconditioned coefficient matrices ${\mathbf{D}}^{-1/2}\tilde{{\mathbf{C}}}{\mathbf{D}}^{-1/2}$ solved with the PCG method and with $k_{S}$ values from 1 to $10^{5}$ (and $k_{O}=1$) are depicted in Fig. 1. It can be observed that the largest eigenvalues decreased with decreasing $k_{O}/k_{S}$ ratios, up to $k_{O}/k_{S}=10^{-2}$ (Fig. 1; Table 1). On the other side of the spectrum, a set of approximately 10,000 small eigenvalues that decrease with decreasing $k_{O}/k_{S}$ ratios can be observed.

**Table 1:** Characteristics of preconditioned (deflated) coefficient matrices, and of PCG and DPCG methods for solving ssSNPBLUP applied to the reduced dataset

$\text{Model}^{\mathrm{a}}$	$\text{Method}^{\mathrm{b}}$	$k_{O}^{\mathrm{c}}$	$k_{S}^{\mathrm{c}}$	$k_{O}/k_{S}$	$\lambda _{min}^{\mathrm{d}}$	$\lambda _{max}^{\mathrm{d}}$	$\kappa ^{\mathrm{e}}$	$\textit{N}^{\mathrm{f}}$
MS	PCG	1	1	1	$1.07\times 10^{-04}$	$1.81\times 10^{2}$	$1.70\times 10^{6}$	1499
MS	PCG	1	2	0.5	$1.07\times 10^{-04}$	$9.11\times 10^{1}$	$8.55\times 10^{5}$	1103
MS	PCG	1	3.3	0.3	$1.07\times 10^{-04}$	$5.51\times 10^{1}$	$5.17\times 10^{5}$	862
MS	PCG	1	$10^{1}$	$10^{-1}$	$1.07\times 10^{-04}$	$1.91\times 10^{1}$	$1.79\times 10^{5}$	560
MS	PCG	1	$10^{2}$	$10^{-2}$	$1.07\times 10^{-04}$	$1.19\times 10^{1}$	$1.12\times 10^{5}$	417
MS	PCG	1	$10^{3}$	$10^{-3}$	$1.06\times 10^{-04}$	$1.19\times 10^{1}$	$1.12\times 10^{5}$	608
MS	PCG	1	$10^{4}$	$10^{-4}$	$4.86\times 10^{-05}$	$1.19\times 10^{1}$	$2.45\times 10^{5}$	1254
MS	PCG	1	$10^{5}$	$10^{-5}$	$4.87\times 10^{-06}$	$1.19\times 10^{1}$	$2.45\times 10^{6}$	2350
MS	PCG	$10^{-1}$	1	$10^{-1}$	$1.07\times 10^{-03}$	$1.91\times 10^{2}$	$1.79\times 10^{5}$	557
MS	PCG	$10^{-2}$	1	$10^{-2}$	$1.07\times 10^{-02}$	$1.19\times 10^{3}$	$1.12\times 10^{5}$	416
MS	PCG	$10^{-3}$	1	$10^{-3}$	$1.06\times 10^{-01}$	$1.19\times 10^{4}$	$1.12\times 10^{5}$	606
MS	PCG	$10^{-4}$	1	$10^{-4}$	$4.86\times 10^{-01}$	$1.19\times 10^{5}$	$2.45\times 10^{5}$	1254
MS	PCG	$10^{-5}$	1	$10^{-5}$	$4.86\times 10^{-01}$	$1.19\times 10^{6}$	$2.45\times 10^{6}$	2367
MS	DPCG (1)	1	1	1	$1.09\times 10^{-04}$	6.44	$5.93\times 10^{4}$	294
MS	DPCG (1)	1	$10^{5}$	$10^{-5}$	$1.09\times 10^{-04}$	6.44	$5.92\times 10^{4}$	293
MS	DPCG (5)	1	1	1	$1.07\times 10^{-04}$	6.44	$6.03\times 10^{4}$	342
MS	DPCG (5)	1	$10^{1}$	$10^{-1}$	$1.07\times 10^{-04}$	6.44	$6.03\times 10^{4}$	331
MS	DPCG (5)	1	$10^{2}$	$10^{-2}$	$1.07\times 10^{-04}$	6.44	$6.04\times 10^{4}$	385
MS	DPCG (5)	1	$10^{3}$	$10^{-3}$	$1.06\times 10^{-04}$	6.44	$6.05\times 10^{4}$	544
MS	DPCG (5)	1	$10^{4}$	$10^{-4}$	$4.96\times 10^{-05}$	6.44	$1.30\times 10^{5}$	961
MS	DPCG (5)	1	$10^{5}$	$10^{-5}$	$4.95\times 10^{-06}$	6.44	$1.30\times 10^{6}$	1456
Liu	PCG	1	1	1	$1.06\times 10^{-04}$	$6.98\times 10^{1}$	$6.56\times 10^{5}$	1401
Liu	PCG	1	$10^{1}$	$10^{-1}$	$1.06\times 10^{-04}$	$1.19\times 10^{1}$	$1.12\times 10^{5}$	561
Liu	PCG	1	$10^{2}$	$10^{-2}$	$1.06\times 10^{-04}$	$1.19\times 10^{1}$	$1.12\times 10^{5}$	563
Liu	PCG	1	$10^{3}$	$10^{-3}$	$5.91\times 10^{-05}$	$1.19\times 10^{1}$	$2.02\times 10^{5}$	1154
Liu	DPCG (5)	1	1	1	$1.07\times 10^{-04}$	6.44	$6.05\times 10^{4}$	419
Liu	DPCG (5)	1	$10^{1}$	$10^{-1}$	$1.07\times 10^{-04}$	6.44	$6.05\times 10^{4}$	399
Liu	DPCG (5)	1	$10^{2}$	$10^{-2}$	$1.06\times 10^{-04}$	6.44	$6.05\times 10^{4}$	520
Liu	DPCG (5)	1	$10^{3}$	$10^{-3}$	$6.02\times 10^{-05}$	6.44	$1.07\times 10^{5}$	1046

${}^{\mathrm{a}}$MS = ssSNPBLUP model proposed by Mantysaari and Stranden [7]; Liu = ssSNPBLUP model proposed by Liu et al. [5]

${}^{\mathrm{b}}$Number of SNP effects per subdomain is within brackets

${}^{\mathrm{c}}$Parameters used for the second-level preconditioner ${\mathbf{D}}$

${}^{\mathrm{d}}$Smallest and largest eigenvalues of the preconditioned (deflated) coefficient matrix

${}^{\mathrm{e}}$Condition number of the preconditioned (deflated) coefficient matrix

${}^{\mathrm{f}}$Number of iterations. A number of iterations equal to 10,000 means that the method failed to converge within 10,000 iterations

**Fig. 2:**
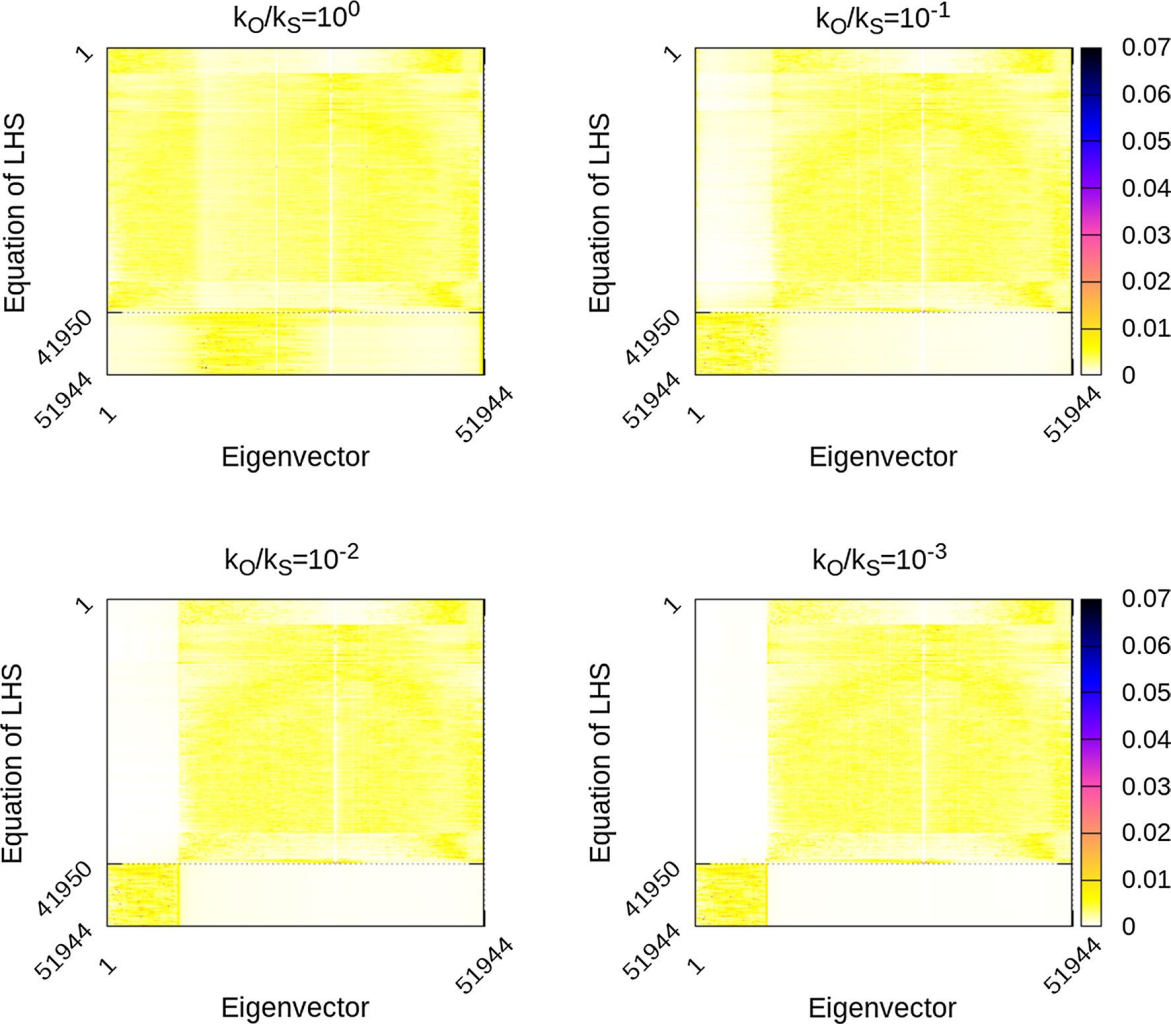
Eigenvectors of preconditioned coefficient matrices with different ratios $k_{O}/k_{S}$ for the reduced dataset. Reported values are aggregate absolute values of sets of 15 eigenvectors sorted following the ascending order of associated eigenvalues, and of 15 entries per eigenvector. Equations associated with SNP effects are from the 41,950th equation until the 51,944th equation

**Fig. 3:**
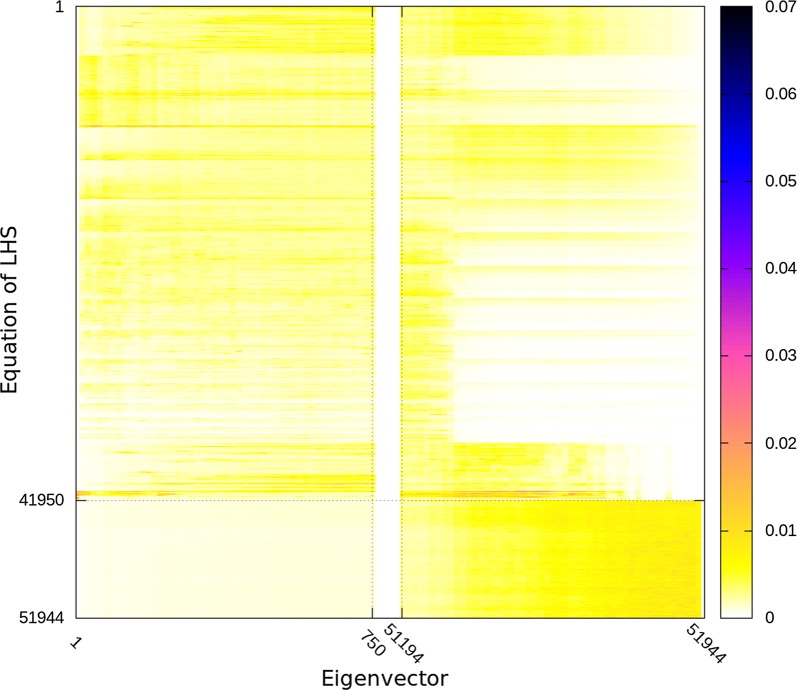
Eigenvectors associated with the 750 smallest and largest eigenvalues of the preconditioned coefficient matrix with the ratio $k_{O}/k_{S}=10^{0}$ for the reduced dataset. Reported values are aggregate absolute values of sets of 15 eigenvectors sorted following the ascending order of associated eigenvalues, and of 15 entries per eigenvector. Darker colors correspond to higher values. Equations associated with SNP effects are from the 41,950th equation until the 51,944th equation

**Fig. 4:**
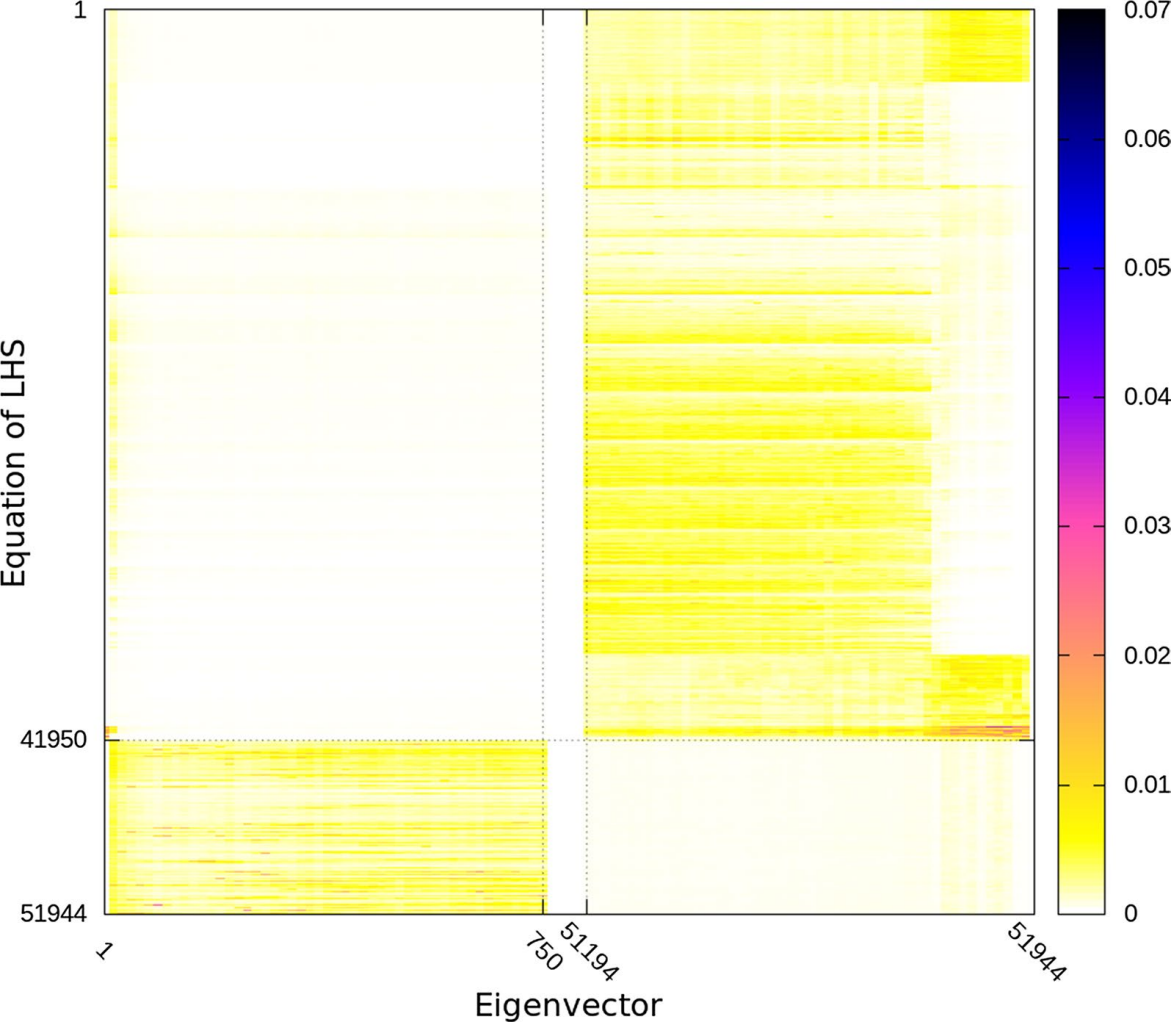
Eigenvectors associated with the 750 smallest and largest eigenvalues of the preconditioned coefficient matrix with the ratio $k_{O}/k_{S}=10^{-2}$ for the reduced dataset. Reported values are aggregate absolute values of sets of 15 eigenvectors sorted following the ascending order of associated eigenvalues, and of 15 entries per eigenvector. Darker colors correspond to higher values. Equations associated with SNP effects are from the 41,950th equation until the 51,944th equation

Figures 2, 3 and 4 depict all the eigenvectors of the ssSNPBLUP_MS preconditioned coefficient matrices ${\mathbf{D}}^{-1/2}\tilde{{\mathbf{C}}}{\mathbf{D}}^{-1/2}$ with different values of $k_{S}$ (and $k_{O}=1$). Non-zero eigenvector entries indicate an association of the eigenvalue (associated with this eigenvector) and the corresponding equations, while (almost) zero entries indicate no such (or a very weak) association. When $k_{O}/k_{S}=1$, it can be observed that the smallest eigenvalues of ${\mathbf{D}}^{-1/2}\tilde{{\mathbf{C}}}{\mathbf{D}}^{-1/2}$ are mainly associated with the equations that are not associated with SNP effects. On the other side, with $k_{O}/k_{S}=1$, the largest eigenvalues of ${\mathbf{D}}^{-1/2}\tilde{{\mathbf{C}}}{\mathbf{D}}^{-1/2}$ are mainly associated with the equations that are associated with SNP effects (Figs. 2 and 3). Decreasing $k_{O}/k_{S}$ ratios resulted in modifying the associations of the extremal eigenvalues (i.e. the smallest and largest eigenvalues) with the equations. Indeed, decreasing $k_{O}/k_{S}$ ratios resulted in the smallest eigenvalues of ${\mathbf{D}}^{-1/2}\tilde{{\mathbf{C}}}{\mathbf{D}}^{-1/2}$ mainly associated with the equations that are associated with SNP effects, and in the largest eigenvalues of ${\mathbf{D}}^{-1/2}\tilde{{\mathbf{C}}}{\mathbf{D}}^{-1/2}$ mainly associated with the equations that are not associated with SNP effects.

**Fig. 5:**
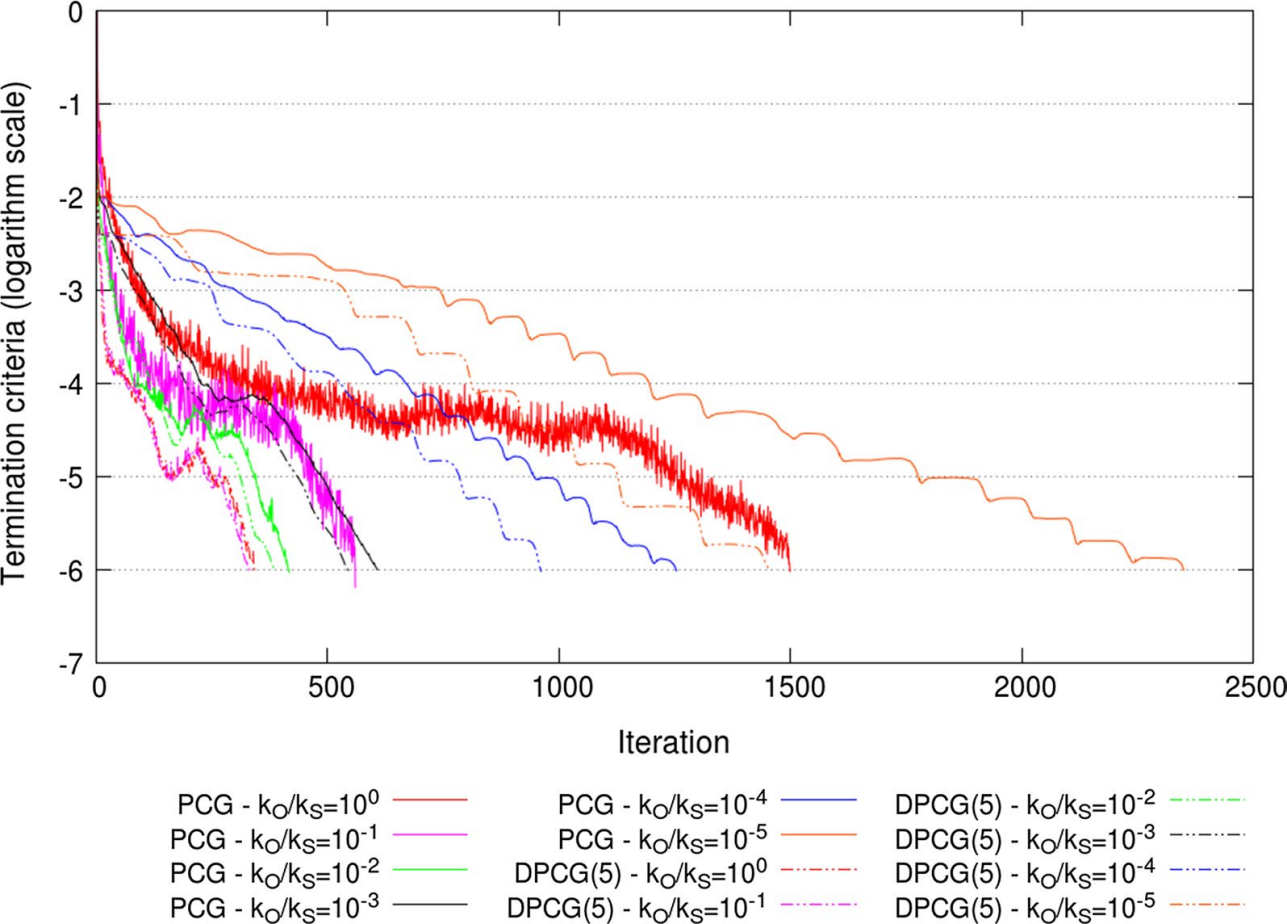
Termination criteria for the reduced dataset for ssSNPBLUP_MS using the PCG and DPCG methods

**Fig. 6:**
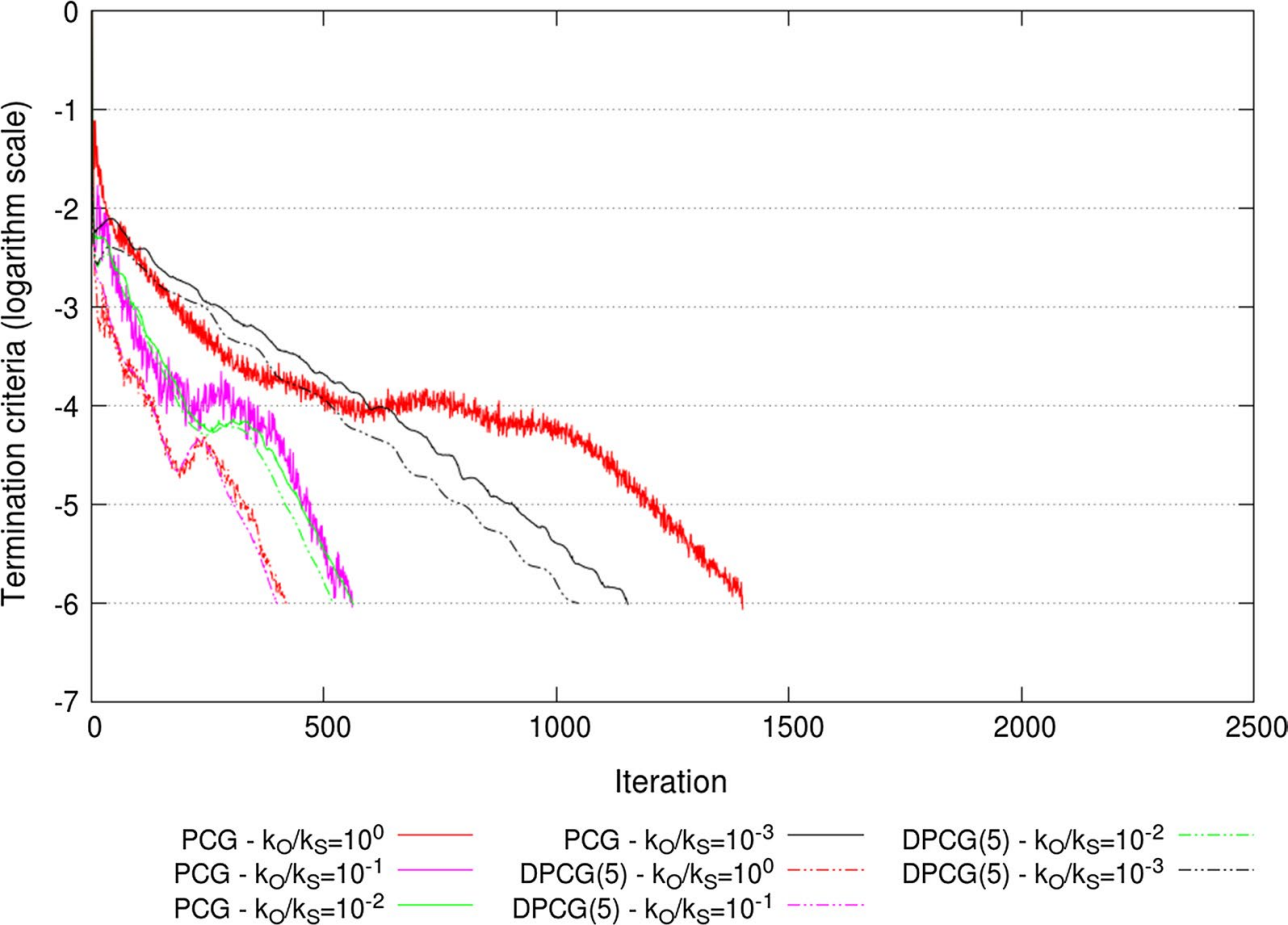
Termination criteria for the reduced dataset for ssSNPBLUP_Liu using the PCG and DPCG methods

The extremal eigenvalues of the ssSNPBLUP_MS and ssSNPBLUP_Liu preconditioned (deflated) coefficient matrices, with various values for $k_{O}$ and $k_{S}$, are in Table 1. For both ssSNPBLUP_MS and ssSNPBLUP_Liu solved with the PCG method, the largest eigenvalues of the preconditioned coefficient matrix decreased with decreasing $k_{O}/k_{S}$ ratios to a lower value of 11.9 that was reached when $k_{O}/k_{S}=10^{-2}$. In addition, for both models, the smallest eigenvalues remained constant with decreasing $k_{O}/k_{S}$ ratios, until $k_{O}/k_{S}=10^{-3}$ for ssSNPBLUP_MS and $k_{O}/k_{S}=10^{-2}$ for ssSNPBLUP_Liu. Due to these results, the effective condition numbers and the number of iterations to reach convergence were the smallest for $k_{O}/k_{S}=10^{-2}$ for ssSNPBLUP_MS and for $k_{O}/k_{S}=10^{-1}$ for ssSNPBLUP_Liu (Table 1; Figs. 5 and 6). In comparison to the PCG method without the second-level preconditioner (i.e., with $k_{O}=k_{S}=1$), the number of iterations to reach convergence decreased by a factor of more than 3.5 for ssSNPBLUP_MS and by a factor of more than 2.4 for ssSNPBLUP_Liu. The minimum number of iterations to reach convergence with the PCG method was 417 for ssSNPBLUP_MS and 561 for ssSNPBLUP_Liu (Table 1; Figs. 5 and 6).

For the same $k_{O}/k_{S}$ ratio, the extremal eigenvalues (i.e. the smallest and largest eigenvalues) of the different preconditioned coefficient matrices were proportional by a factor of $k_{O}^{-1}$ (Table 1). Therefore, for the same $k_{O}/k_{S}$ ratio the effective condition numbers of the different preconditioned coefficient matrices and the associated numbers of equations to reach convergence were the same (Table 1). It is also worth noting that, for a fixed value of $k_{O}$, the largest eigenvalues decreased almost proportionally by a factor of $k_{S}^{-1}$ with decreasing $k_{O}/k_{S}$ ratios until they reached their lower bound (Table 1).

For both ssSNPBLUP_MS and ssSNPBLUP_Liu solved with the DPCG method and 5 SNPs per subdomain, the largest eigenvalues of the preconditioned deflated coefficient matrices remained constant (around 6.44) for all $k_{O}/k_{S}$ ratios (Table 1). However, for both models, the smallest eigenvalues started to decrease for $k_{O}/k_{S}$ ratios smaller than $10^{-3}$ ($10^{-2}$) for ssSNPBLUP_MS (ssSNPBLUP_Liu). These unfavourable decreases of the smaller eigenvalues with decreasing $k_{O}/k_{S}$ ratios resulted in increasing the effective condition numbers and the number of iterations to reach convergence when the second-level preconditioner ${\mathbf{D}}$ was applied with the DPCG method (Table 1; Figs. 5 and 6).

### Field dataset

For the field dataset, regarding the extremal eigenvalues, the application of the second-level preconditioner ${\mathbf{D}}$ together with the PCG method led to a decrease of the largest eigenvalues of the preconditioned coefficient matrix from $1.8\times 10^{3}$ for ssSNPBLUP_MS, and from $1.4\times 10^{2}$ for ssSNPBLUP_Liu, to about 5. Ratios of $k_{O}/k_{S}$ smaller than $10^{-3}$ for ssSNPBLUP_MS and smaller than $10^{-2}$ for ssSNPBLUP_Liu did not further change the largest eigenvalues (Table 2). For the DPCG method applied to ssSNPBLUP_MS, the largest eigenvalues of the preconditioned deflated coefficient matrices remained constant for all $k_{O}/k_{S}$ ratios (Table 2). For the DPCG method applied to ssSNPBLUP_Liu, the largest eigenvalues of the preconditioned deflated coefficient matrices slightly decreased with $k_{O}/k_{S}=10^{-1}$ and then remained constant for all $k_{O}/k_{S}$ ratios (Table 2). The application of the second-level preconditioner ${\mathbf{D}}$ with both the PCG and DPCG methods led to the smallest eigenvalues of the preconditioned (deflated) coefficient matrices decreasing with decreasing $k_{O}/k_{S}$ ratios (Table 2).

**Table 2:** Characteristics of preconditioned (deflated) coefficient matrices, and of PCG and DPCG methods for solving ssSNPBLUP applied to the field dataset

$\text{Model}^{\mathrm{a}}$	Method	$k_{O}/k_{S}^{\mathrm{b}}$	$\lambda _{min}^{\mathrm{c}}$	$\lambda _{max}^{\mathrm{c}}$	$\kappa ^{\mathrm{d}}$	$\textit{N}^{\mathrm{e}}$	$\text{Iterative time}^{\mathrm{f}}$	$\text{Time/iter.}^{\mathrm{g}}$	$\text{Total time}^{\mathrm{h}}$
MS	PCG	1	$3.70\times 10^{-5}$	$1.75\times 10^{3}$	$4.74\times 10^{7}$	10,000	44,808	4.5	46,081
MS	PCG	$10^{-1}$	$1.18\times 10^{-5}$	$1.77\times 10^{2}$	$1.51\times 10^{7}$	10,000	51,768	5.2	53,550
MS	PCG	$10^{-2}$	$4.37\times 10^{-6}$	$1.95\times 10^{1}$	$4.45\times 10^{6}$	6210	34,139	5.5	35,812
MS	PCG	$10^{-3}$	$3.99\times 10^{-6}$	5.08	$1.27\times 10^{6}$	3825	19,043	5.0	20,866
MS	PCG	$10^{-4}$	$1.50\times 10^{-6}$	5.07	$3.37\times 10^{6}$	7336	54,326	7.4	56,475
MS	DPCG	1	$2.86\times 10^{-5}$	4.77	$1.67\times 10^{5}$	748	6527	8.7	17,229
MS	DPCG	$10^{-1}$	$1.41\times 10^{-5}$	4.77	$3.37\times 10^{5}$	1211	11,864	9.8	22,947
MS	DPCG	$10^{-2}$	$9.17\times 10^{-6}$	4.77	$5.20\times 10^{5}$	1778	17,030	9.6	28,615
MS	DPCG	$10^{-3}$	$7.50\times 10^{-6}$	4.77	$6.36\times 10^{5}$	2569	23,676	9.2	35,497
Liu	PCG	1	$7.38\times 10^{-6}$	$1.43\times 10^{2}$	$1.93\times 10^{7}$	10,000	44,122	4.4	45,083
Liu	PCG	$10^{-1}$	$3.66\times 10^{-6}$	$1.52\times 10^{1}$	$4.14\times 10^{6}$	6049	31,085	5.1	32,018
Liu	PCG	$10^{-2}$	$4.29\times 10^{-6}$	5.07	$1.18\times 10^{6}$	2669	13,225	5.0	13,888
Liu	PCG	$10^{-3}$	$3.51\times 10^{-6}$	5.07	$1.44\times 10^{6}$	3606	20,578	5.7	21,458
Liu	PCG	$10^{-4}$	$1.69\times 10^{-6}$	5.07	$3.00\times 10^{6}$	7033	33,534	4.8	34,675
Liu	DPCG	1	$5.40\times 10^{-6}$	5.31	$9.85\times 10^{5}$	2877	22,791	7.9	26,521
Liu	DPCG	$10^{-1}$	$6.91\times 10^{-6}$	4.77	$6.90\times 10^{5}$	1628	14,231	8.7	18,049
Liu	DPCG	$10^{-2}$	$5.23\times 10^{-6}$	4.77	$9.11\times 10^{5}$	2234	23,244	10.4	28,057
Liu	DPCG	$10^{-3}$	$4.31\times 10^{-6}$	4.77	$1.11\times 10^{6}$	3106	34,950	11.3	39,603

${}^{\mathrm{a}}$MS = ssSNPBLUP model proposed by Mantysaari and Stranden [7]; Liu = ssSNPBLUP model proposed by Liu et al. [5];

${}^{\mathrm{b}}$Parameters used for the second-level preconditioner;

${}^{\mathrm{c}}$Smallest and largest eigenvalues of the preconditioned (deflated) coefficient matrix;

${}^{\mathrm{d}}$Condition number of the preconditioned (deflated) coefficient matrix;

${}^{\mathrm{e}}$Number of iterations. A number of iterations equal to 10,000 means that the method failed to converge within 10,000 iterations;

${}^{\mathrm{f}}$Wall clock time (seconds) for the iterative process;

${}^{\mathrm{g}}$Average wall clock time (seconds) per iteration;

${}^{\mathrm{h}}$Wall clock time (seconds) for a complete process (including I/O operations)

**Fig. 7:**
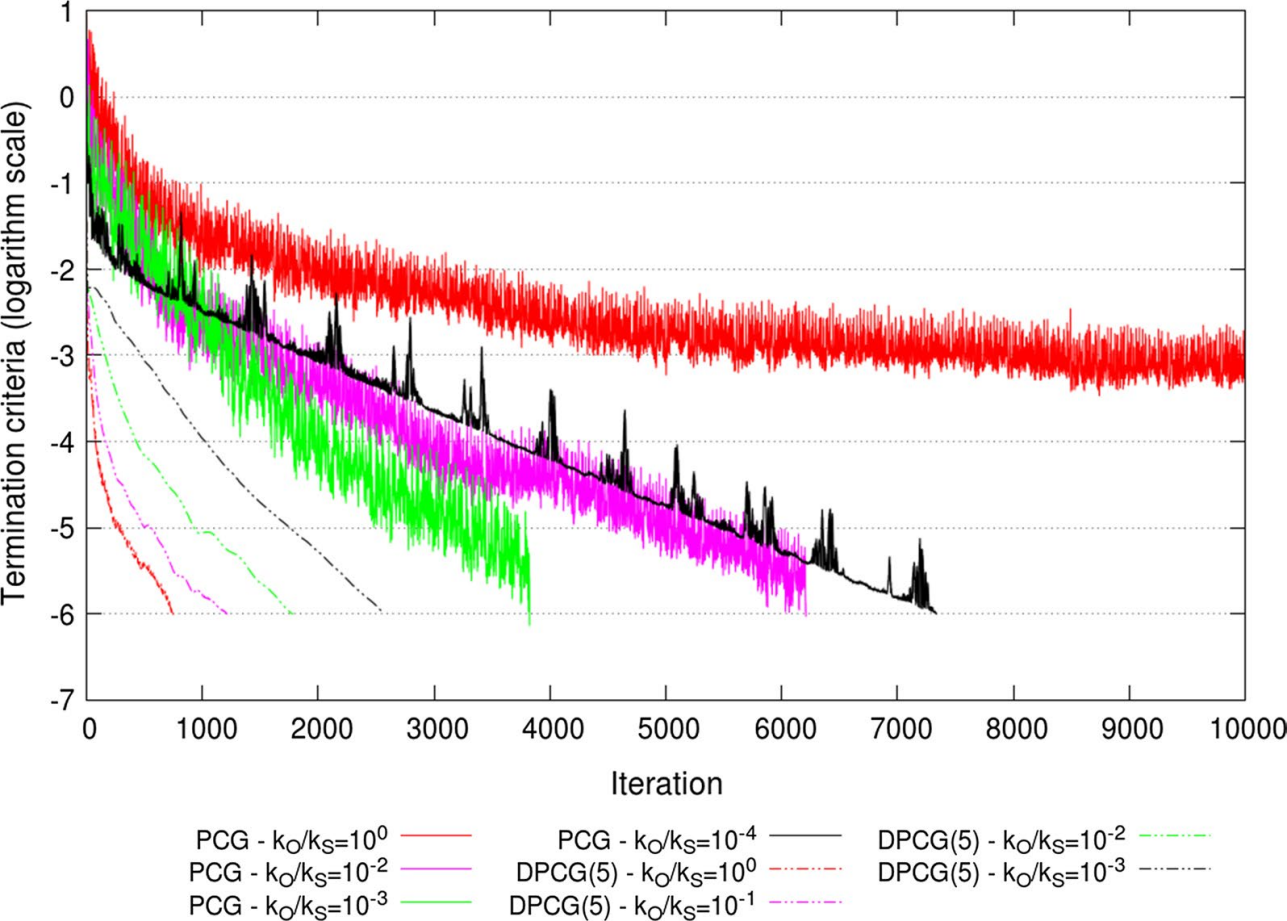
Termination criteria for the field dataset for ssSNPBLUP_MS using the PCG and DPCG methods

**Fig. 8:**
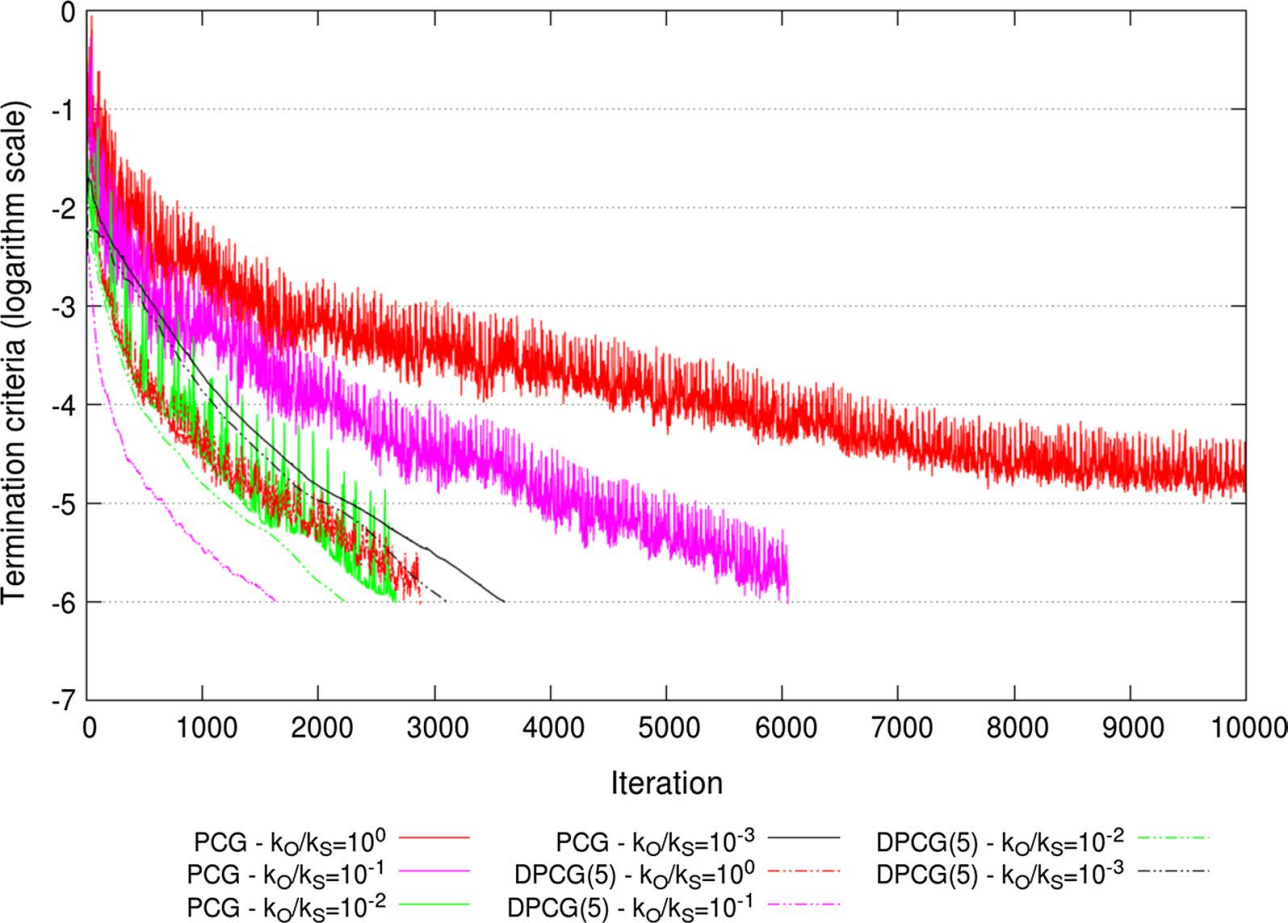
Termination criteria for the field dataset for ssSNPBLUP_Liu using the PCG and DPCG methods

These observed patterns of extremal eigenvalues resulted in an optimal $k_{O}/k_{S} = 10^{-3}$ ratio for the PCG method applied to ssSNPBLUP_MS and an optimal $k_{O}/k_{S} = 10^{-2}$ ratio for the PCG method applied to ssSNPBLUP_Liu, in terms of effective condition numbers and numbers of iterations to reach convergence (Table 2; Figs. 7 and 8). With these ratios, the PCG method converged within 3825 iterations for ssSNPBLUP_MS and within 2665 iterations for ssSNPBLUP_Liu, while the PCG method did not converge within 10,000 iterations for both models (Table 2; Figs. 7 and 8). For the DPCG method, the application of the second-level preconditioner ${\mathbf{D}}$ generally deteriorated the effective condition numbers and numbers of iterations to reach convergence, for both ssSNPBLUP_MS and ssSNPBLUP_Liu. The DPCG method converged within 748 iterations for ssSNPBLUP_MS with $k_{O}/k_{S}=1$ and within 2877 iterations for ssSNPBLUP_Liu with $k_{O}/k_{S}=10^{-1}$ (Table 2; Figs. 7 and 8).

The total wall clock times of the iterative processes and for the complete processes (including I/O operations and computation of the preconditioners, and Galerkin matrices) for the PCG and DPCG methods are in Table 2. Across all combinations of systems of equations and solvers, the smallest wall clock time for the complete process was approximately 14,000 s for the PCG method with the second-level preconditioner ${\mathbf{D}}$ applied to ssSNPBLUP_Liu. Slightly greater wall clock times were needed for ssSNPBLUP_MS solved with the DPCG method (without the second-level preconditioner ${\mathbf{D}}$). It is worth noting that the wall clock times needed for the computation of the inverse of the Galerkin matrix (${\mathbf{E}}^{-1}$) were approximately 9700 s for ssSNPBLUP_MS and approximately 2500 s for ssSNPBLUP_Liu.

## Discussion

In this study, we introduced a second-level diagonal preconditioner ${\mathbf{D}}$ that results in smaller effective condition numbers of the preconditioned (deflated) coefficient matrices and in improved convergence patterns for two different ssSNPBLUP mixed model equations. From the theory and based on the results, the use of the second-level preconditioner ${\mathbf{D}}$ results in improved effective condition numbers of the preconditioned (deflated) coefficient matrices of ssSNPBLUP by decreasing the largest eigenvalues, while the smallest eigenvalues remain constant, or decrease at a lower rate than the largest eigenvalues. In this section, we will discuss the following three points: (1) the influence of the second-level diagonal preconditioner ${\mathbf{D}}$ on the eigenvalues and associated eigenvectors of the preconditioned (deflated) coefficient matrices of ssSNPBLUP; (2) the application of the second-level preconditioner in ssSNPBLUP evaluations; and (3) the possible application of the second-level preconditioner ${\mathbf{D}}$ to more complex ssSNPBLUP models and to models other than ssSNPBLUP.

### Influence of ${\mathbf{D}}$ on the eigenvalues and associated eigenvectors

Applying the second-level preconditioner ${\mathbf{D}}$ with an optimal $k_{O}/k_{S}$ ratio to the linear systems of ssSNPBLUP results in a decrease of the largest eigenvalues of the preconditioned (deflated) coefficient matrices of ssSNPBLUP. As observed by Vandenplas et al. [4] and in comparison with ssGBLUP, the largest eigenvalues that influence the convergence of the PCG method applied to ssSNPBLUP_MS were associated with SNP effects. The second-level preconditioner ${\mathbf{D}}$ allows a decrease of these largest eigenvalues by multiplying all entries of these SNP equations of the preconditioned coefficient matrices by a value proportional to $k_{O}/k_{S}$, as shown with the Gershgorin circle algorithm [18] [see Eq. (4)]. However, if the $k_{O}/k_{S}$ ratio is applied to a set of equations that are not associated with the largest eigenvalues of the preconditioned (deflated) coefficient matrices, the second-level preconditioner ${\mathbf{D}}$ will not result in decreased largest eigenvalues. This behaviour was observed when the second-level preconditioner ${\mathbf{D}}$ was applied to ssSNPBLUP_MS with the DPCG method for the reduced dataset (Table 1). For these scenarios, the DPCG method already annihilated all the largest unfavourable eigenvalues up to the lower bound of the largest eigenvalue that is allowed with the second-level preconditioner ${\mathbf{D}}$. Therefore, the second-level preconditioner ${\mathbf{D}}$ did not further decrease the largest eigenvalues. It is worth noting that, if the DPCG method did not annihilate all the unfavourable largest eigenvalues up to the lower bound defined by Eq. (4), the application of the second-level preconditioner ${\mathbf{D}}$ with the DPCG method did remove these remaining largest eigenvalues, as shown by the results for ssSNPBLUP_Liu applied to the field dataset (Table 2).

The decrease of the largest eigenvalues of the preconditioned coefficient matrices with decreasing $k_{O}/k_{S}$ ratios (and until the lower bound is reached) can be explained by the sparsity pattern of the eigenvectors associated with the largest eigenvalues of the preconditioned coefficient matrices $\tilde{{\mathbf{C}}}$ of ssSNPBLUP. Indeed, Figs. 2 and 3 show that the entries that correspond to the equations that are not associated with SNP effects, are close to 0 for the eigenvectors associated with the largest eigenvalues of $\tilde{{\mathbf{C}}}$ of ssSNPBLUP_MS. Accordingly, if we assume that these entries are 0, i.e.,

$$\tilde{{\mathbf{v}}}_{max} = \left[ \begin{array}{c} \tilde{{\mathbf{v}}}_{O_{max}} \\ \tilde{{\mathbf{v}}}_{S_{max}} \end{array} \right] = \left[ \begin{array}{c} {\mathbf{0}} \\ \tilde{{\mathbf{v}}}_{S_{max}} \end{array} \right]$$

being an eigenvector associated with one of largest eigenvalues of $\tilde{{\mathbf{C}}}$, it follows that the largest eigenvalues of $\tilde{{\mathbf{C}}}$ multiplied by $k_{S}^{-1}$ are also the eigenvalues of ${\mathbf{D}}^{-1/2}\tilde{{\mathbf{C}}}{\mathbf{D}}^{-1/2}$. These largest eigenvalues of $\tilde{{\mathbf{C}}}$ will therefore be equal to the largest eigenvalues of ${\mathbf{D}}^{-1/2}\tilde{{\mathbf{C}}}{\mathbf{D}}^{-1/2}$ until the lower bound defined by Eq. (4) is reached (see Additional file 2 for the derivation). This observation can also motivate an educated guess for an optimal $k_{O}/k_{S}$ ratio for ssSNPBLUP with one additive genetic effect. If the largest eigenvalues $\lambda _{max}\left( \tilde{{\mathbf{C}}}\right)$ and $\lambda _{max}\left( \tilde{{\mathbf{C}}}_{OO}\right)$ are (approximately) known, an educated guess for the $k_{O}/k_{S}$ ratio can be equal to $\frac{k_{O}}{k_{S}} = k_{O}\frac{\lambda _{max}\left( \tilde{{\mathbf{C}}}_{OO}\right) }{\lambda _{max}\left( \tilde{{\mathbf{C}}}\right) }$. For example, in our cases, $\lambda _{max}\left( \tilde{{\mathbf{C}}}_{OO}\right)$ was always equal to the largest eigenvalue of the preconditioned coefficient matrix of a pedigree BLUP (results not shown). It follows that the educated guess for the field dataset is equal to $3.0\times 10^{-3}$ for ssSNPBLUP_MS and $3.5\times 10^{-2}$ for ssSNPBLUP_Liu, since $\lambda _{max}\left( \tilde{{\mathbf{C}}}_{OO}\right) =5.07$. Both values are of the same order as the corresponding optimal $k_{O}/k_{S}$ ratios. However, the second-level preconditioner ${\mathbf{D}}$ will be effective only if the smallest eigenvalues of the preconditioned coefficient matrices are not influenced, or at least less influenced than the largest eigenvalues, by the second-level preconditioner ${\mathbf{D}}$.

The decrease of the smallest eigenvalues of the preconditioned (deflated) coefficient matrices mainly depends on the sparsity pattern of the eigenvectors associated with the smallest eigenvalues. We formulated a sufficient condition such that the smallest eigenvalues remain constant when the second-level preconditioner is applied. While this sufficient condition is not fulfilled for the reduced dataset (and probably also not for the field dataset), it can help us to predict the behaviour of the smallest eigenvalues based on the sparsity pattern of the associated eigenvectors. For example, if the eigenvector associated with the smallest eigenvalue of $\tilde{{\mathbf{C}}}$ has mainly non-zero entries corresponding to the equations associated with SNP effects, the use of the second-level preconditioner ${\mathbf{D}}$ will most likely result in a decrease of the smallest eigenvalues proportional to $k_{S}^{-1}$, which is undesirable. Other behaviours of the smallest eigenvalues of the preconditioned (deflated) coefficient matrices can lead to the conclusion that the associated eigenvectors have a different sparsity pattern, which helps understand if and how the use of the proposed second-level diagonal preconditioner will be beneficial.

### Application of ${\mathbf{D}}$ in ssSNPBLUP evaluations

The second-level preconditioner ${\mathbf{D}}$ is easy to implement in existing software and does not influence the computational costs of a PCG iteration, since it can be merged with the preconditioner ${\mathbf{M}}$. Indeed, it is sufficient to multiply the entries of ${\mathbf{M}}^{-1}$ that correspond to the equations associated with SNP effects by an optimal $k_{O}/k_{S}$ ratio to implement the second-level preconditioner ${\mathbf{D}}$. Furthermore, the value of an optimal $k_{O}/k_{S}$ ratio for a ssSNPBLUP evaluation can be determined by testing a range of values around the educated guess defined previously and then re-used for several subsequent ssSNPBLUP evaluations, because additional data for each new evaluation is only a fraction of the data previously used and will therefore not modify, or will modify only slightly, the properties of the preconditioned coefficient matrices $\tilde{{\mathbf{C}}}$.

In this study, we used the second-level diagonal preconditioner ${\mathbf{D}}$ for two different ssSNPBLUP models. To our knowledge, it is the first time that ssSNPBLUP_Liu was successfully applied until convergence with real datasets [3, 5]. From our results, it seems that the preconditioned coefficient matrices of ssSNPBLUP_Liu are better conditioned than the preconditioned coefficient matrices of ssSNPBLUP_MS, leading to better convergence patterns for ssSNPBLUP_Liu. Therefore, among all possible combinations of linear systems (i.e., ssSNPBLUP_MS and ssSNPBLUP_Liu), solvers (i.e., the PCG and DPCG methods) and the application (or not) of the second-level preconditioner ${\mathbf{D}}$, it seems that ssSNPBLUP_Liu solved with the PCG method combined with the second-level preconditioner ${\mathbf{D}}$ is the most efficient in terms of total wall clock times and implementation. However, in our study it was tested only on two datasets and the most efficient combination of linear system and solver will most likely be situation-dependent.

### Application of ${\mathbf{D}}$ to other scenarios

The proposed second-level preconditioner ${\mathbf{D}}$ can be applied and may be beneficial for ssSNPBLUP models that involve multiple additive genetic effects, or for other models that include an effect that would result in an increase to the largest eigenvalues of the preconditioned coefficient matrices. The developed theory does not require a multivariate ssSNPBLUP with only one additive genetic effect. As such, for example, if multiple additive genetic effects are fitted into the ssSNPBLUP model, such as direct and maternal genetic effects, the second-level preconditioner ${\mathbf{D}}$ could be used with different $k_{O}/k_{S}$ ratios applied separately to the direct and maternal SNP effects. A similar strategy was successfully applied for ssSNPBLUP proposed by Fernando et al. [2] with French beef cattle datasets (Thierry Tribout, personal communication). Furthermore, the second-level preconditioner ${\mathbf{D}}$ could be used to improve the convergence pattern of models other than ssSNPBLUP. For example, with the field dataset, the addition of the genetic groups fitted explicitly as random covariables in the model for pedigree-BLUP (that is, without genomic information) led to an increase of the largest eigenvalue of the preconditioned coefficient matrix from 5.1 to 14.8. The introduction of the second-level preconditioner ${\mathbf{D}}$ into the preconditioned linear system of pedigree-BLUP with a $k_{O}/k_{S}=10^{-1}$ ratio applied to the equations associated with the genetic groups reduced the largest eigenvalues to 6.0, resulting in a decrease of the effective condition number by a factor of 2.6. This decrease of the effective condition number translated to a decrease in the number of iterations to reach convergence from 843 to 660.

## Conclusions

The proposed second-level preconditioner ${\mathbf{D}}$ is easy to implement in existing software and can improve the convergence of the PCG and DPCG methods applied to different ssSNPBLUP methods. Based on our results, the ssSNPBLUP system of equations proposed by Liu et al. [5] solved using the PCG method and the second-level preconditioner seems to be most efficient. However, the optimal combination of ssSNPBLUP and solver will most likely be situation-dependent.

## Additional files

**Additional file 1.** Bounds of the largest eigenvalue of the preconditioned coefficient matrix of ssSNPBLUP Derivation of the lower and upper bounds of the largest eigenvalue of the preconditioned coefficient matrix of ssSNPBLUP.

**Additional file 2.** Proof of the sufficient condition Proof of the sufficient condition.
