# Supplementary material for: A second-level diagonal preconditioner for single-step SNPBLUP
Source: Genet Sel Evol. 2019 Jun 25;51:30. doi: 10.1186/s12711-019-0472-8 (PMC6593613; doi:10.1186/s12711-019-0472-8)
Supplement: Supplementary file 1 — Additional file 1. Bounds of the largest eigenvalue of the preconditioned coefficient matrix of ssSNPBLUP Derivation of the lower and upper bounds of the largest eigenvalue of the preconditioned coefficient matrix of ssSNPBLUP. [file 12711_2019_472_MOESM1_ESM.pdf]

Additional file 1: Bounds of the largest eigenvalue of the preconditioned coefficient matrix of ssSNPBLUP

Here we derive the lower and upper bounds of the largest eigenvalue of the preconditioned coefficient matrix of ssSNPBLUP.

Let the matrix $\mathbf{C}$ be a coefficient matrix of ssSNPBLUP.

Let the matrix $\mathbf{M}$ be a (block-)diagonal preconditioner associated with $\mathbf{C}$.

Let the matrix $\tilde{\mathbf{C}}$ be a preconditioned coefficient matrix defined as $\tilde{\mathbf{C}} = \mathbf{M}^{-1/2}\mathbf{C}\mathbf{M}^{-1/2}$. The matrix $\tilde{\mathbf{C}}$ can be partitioned between equations not associated with (O) and associated with (S) SNP effects as:

$$\tilde{\mathbf{C}} = \begin{bmatrix} \tilde{\mathbf{C}}_{OO} & \tilde{\mathbf{C}}_{OS} \\ \tilde{\mathbf{C}}_{SO} & \tilde{\mathbf{C}}_{SS} \end{bmatrix}.$$

Let the matrix $\mathbf{D}$ be a diagonal matrix defined as

$$\mathbf{D} = \begin{bmatrix} k_O\mathbf{I}_{OO} & \mathbf{0} \\ \mathbf{0} & k_S\mathbf{I}_{SS} \end{bmatrix}$$

where $\mathbf{I}_{OO}$ is an identity matrix of size equal to the number of equations that are not associated with the SNP effects, $\mathbf{I}_{SS}$ is an identity matrix of size equal to the number of equations that are associated with the SNP effects, and k_O and k_S are real positive numbers.

From the Gershgorin circle theorem, it follows that the largest eigenvalue of the preconditioned coefficient matrix $\mathbf{D}^{-1/2}\tilde{\mathbf{C}}\mathbf{D}^{-1/2}$ is bounded by, for all i and j:

$$\lambda_{max}\left(\mathbf{D}^{-1/2}\tilde{\mathbf{C}}\mathbf{D}^{-1/2}\right) \leq \max_i \left\{ \mathbf{D}_{ii}^{-1/2}\tilde{\mathbf{C}}_{ii}\mathbf{D}_{ii}^{-1/2} + \sum_{j\neq i} |\mathbf{D}_{ii}^{-1/2}\tilde{\mathbf{C}}_{ij}\mathbf{D}_{jj}^{-1/2}| \right\} \quad (1)$$

Partitioned between the equations associated with SNP effects (S) and with the other effects (O), the preconditioned coefficient matrix $\mathbf{D}^{-1/2}\tilde{\mathbf{C}}\mathbf{D}^{-1/2}$ is equal to:

$$\begin{aligned} \mathbf{D}^{-1/2}\tilde{\mathbf{C}}\mathbf{D}^{-1/2} &= \begin{bmatrix} k_O\mathbf{I}_{OO} & \mathbf{0} \\ \mathbf{0} & k_S\mathbf{I}_{SS} \end{bmatrix}^{-1/2} \begin{bmatrix} \tilde{\mathbf{C}}_{OO} & \tilde{\mathbf{C}}_{OS} \\ \tilde{\mathbf{C}}_{SO} & \tilde{\mathbf{C}}_{SS} \end{bmatrix} \begin{bmatrix} k_O\mathbf{I}_{OO} & \mathbf{0} \\ \mathbf{0} & k_S\mathbf{I}_{SS} \end{bmatrix}^{-1/2} \\ &= k_O^{-1} \begin{bmatrix} \tilde{\mathbf{C}}_{OO} & \sqrt{\frac{k_O}{k_S}}\tilde{\mathbf{C}}_{OS} \\ \sqrt{\frac{k_O}{k_S}}\tilde{\mathbf{C}}_{SO} & \frac{k_O}{k_S}\tilde{\mathbf{C}}_{SS} \end{bmatrix} \end{aligned}$$

With this partitioning, it follows that:

$$\begin{aligned}\lambda_{max}\left(\mathbf{D}^{-1/2}\tilde{\mathbf{C}}\mathbf{D}^{-1/2}\right) &\leq \underset{i}{max}\left\{\mathbf{D}_{ii}^{-1/2}\tilde{\mathbf{C}}_{ii}\mathbf{D}_{ii}^{-1/2}+\sum_{j\neq i}|\mathbf{D}_{ii}^{-1/2}\tilde{\mathbf{C}}_{ij}\mathbf{D}_{jj}^{-1/2}|\right\}\\ &\leq k_O^{-1}max\left\{a,b\right\}\end{aligned}$$

with $a=\underset{k}{max}\left\{\tilde{\mathbf{C}}_{OO_{kk}}+\sum_{j\neq k}|\tilde{\mathbf{C}}_{OO_{kj}}|+\sqrt{\frac{k_O}{k_S}}\sum_{j\neq k}|\tilde{\mathbf{C}}_{OS_{kj}}|\right\}$,
$b=\underset{l}{max}\left\{\frac{k_O}{k_S}\tilde{\mathbf{C}}_{SS_{ll}}+\frac{k_O}{k_S}\sum_{j\neq l}|\tilde{\mathbf{C}}_{SS_{lj}}|+\sqrt{\frac{k_O}{k_S}}\sum_{j\neq l}|\tilde{\mathbf{C}}_{SO_{lj}}|\right\}$, and k and l referring to the equations not associated with and associated with the SNP effects, respectively.

For a fixed value of k_O, if $\frac{k_O}{k_S}=0$, the largest eigenvalue of $\mathbf{D}^{-1/2}\tilde{\mathbf{C}}\mathbf{D}^{-1/2}$ is equal to the largest eigenvalue of $k_O^{-1}\tilde{\mathbf{C}}_{OO}$. Therefore, $\lambda_{max}\left(\mathbf{D}^{-1/2}\tilde{\mathbf{C}}\mathbf{D}^{-1/2}\right)$ has the following lower and upper bounds:

$$k_O^{-1}\lambda_{max}\left(\tilde{\mathbf{C}}_{OO}\right)\leq\lambda_{max}\left(\mathbf{D}^{-1/2}\tilde{\mathbf{C}}\mathbf{D}^{-1/2}\right)\leq k_O^{-1}max\left\{a,b\right\}$$
