# Supplementary material for: A second-level diagonal preconditioner for single-step SNPBLUP
Source: Genet Sel Evol. 2019 Jun 25;51:30. doi: 10.1186/s12711-019-0472-8 (PMC6593613; doi:10.1186/s12711-019-0472-8)
Supplement: Supplementary file 2 — Additional file 2. Proof of the sufficient condition Proof of the sufficient condition. [file 12711_2019_472_MOESM2_ESM.pdf]

Additional file 2: Proof of the sufficient condition

Let the matrix $\mathbf{C}$ be a coefficient matrix of ssSNPBLUP.

Let the matrix $\mathbf{M}$ be a (block-)diagonal preconditioner associated with $\mathbf{C}$.

Let the matrix $\tilde{\mathbf{C}}$ be a preconditioned coefficient matrix defined as $\tilde{\mathbf{C}} = \mathbf{M}^{-1/2}\mathbf{C}\mathbf{M}^{-1/2}$. The matrix $\tilde{\mathbf{C}}$ can be partitioned between equations not associated with (O) and associated with (S) SNP effects as:

$$\tilde{\mathbf{C}} = \begin{bmatrix} \tilde{\mathbf{C}}_{OO} & \tilde{\mathbf{C}}_{OS} \\ \tilde{\mathbf{C}}_{SO} & \tilde{\mathbf{C}}_{SS} \end{bmatrix}.$$

Let the matrix $\mathbf{D}$ be a diagonal matrix defined as

$$\mathbf{D} = \begin{bmatrix} k_O\mathbf{I}_{OO} & \mathbf{0} \\ \mathbf{0} & k_S\mathbf{I}_{SS} \end{bmatrix}$$

where $\mathbf{I}_{OO}$ is an identity matrix of size equal to the number of equations that are not associated with the SNP effects, $\mathbf{I}_{SS}$ is an identity matrix of size equal to the number of equations that are associated with the SNP effects, and k_O and k_S are real positive numbers.

Let the matrix $\tilde{\mathbf{V}}$ be a matrix containing (columnwise) all the eigenvectors of $\tilde{\mathbf{C}}$ sorted following the ascending order of their associated eigenvalues. The matrix $\tilde{\mathbf{V}}$ can be partitioned into a matrix $\tilde{\mathbf{V}}_1$ storing eigenvectors associated with eigenvalues at the left-hand side of the spectrum of $\tilde{\mathbf{C}}$ (that includes the smallest eigenvalues) and a matrix $\tilde{\mathbf{V}}_2$ storing eigenvectors at the right-hand side of the spectrum of $\tilde{\mathbf{C}}$ (that includes the largest eigenvalues), and between equations not associated with and associated with SNP effects, as follows: $\tilde{\mathbf{V}} = \begin{bmatrix} \tilde{\mathbf{V}}_1 & \tilde{\mathbf{V}}_2 \end{bmatrix} = \begin{bmatrix} \tilde{\mathbf{V}}_{O1} & \tilde{\mathbf{V}}_{O2} \\ \tilde{\mathbf{V}}_{S1} & \tilde{\mathbf{V}}_{S2} \end{bmatrix}$.

Sufficient condition

A sufficient condition to ensure that $\lambda_{min}\left(\mathbf{D}^{-1/2}\tilde{\mathbf{C}}\mathbf{D}^{-1/2}\right) = k_O^{-1}\lambda_{min}\left(\tilde{\mathbf{C}}\right)$, is that $\tilde{\mathbf{V}}_{S1} = \mathbf{0}$, $\tilde{\mathbf{V}}_{O2} = \mathbf{0}$, and that all eigenvalues associated with an eigenvector of $\tilde{\mathbf{V}}_2$ are equal to, or larger than, $\frac{k_S}{k_O}\lambda_{min}\left(\tilde{\mathbf{C}}\right)$.

Proof

This sufficient condition is proven in three steps.

First, if $\tilde{\mathbf{V}}_{S1} = \mathbf{0}$, it implies that each ith eigenvalue associated with an eigenvector of $\tilde{\mathbf{V}}_1$, that is $\lambda_{1_i}\left(\tilde{\mathbf{C}}\right)$, is proportional by a factor k_O to an eigenvalue of $\mathbf{D}^{-1/2}\tilde{\mathbf{C}}\mathbf{D}^{-1/2}$, that is $\lambda_i\left(\mathbf{D}^{-1/2}\tilde{\mathbf{C}}\mathbf{D}^{-1/2}\right) = k_O^{-1}\lambda_{1_i}\left(\tilde{\mathbf{C}}\right)$, because:

$$
\begin{aligned}
\tilde{\mathbf{C}}\tilde{\mathbf{v}}_{1_i} = \lambda_{1_i}\left(\tilde{\mathbf{C}}\right)\tilde{\mathbf{v}}_{1_i} \quad &\Longleftrightarrow \quad \mathbf{D}^{-1/2}\tilde{\mathbf{C}}\tilde{\mathbf{v}}_{1_i} = \lambda_{1_i}\left(\tilde{\mathbf{C}}\right)\mathbf{D}^{-1/2}\tilde{\mathbf{v}}_{1_i} \\
&\Longleftrightarrow \quad \mathbf{D}^{-1/2}\tilde{\mathbf{C}}\mathbf{D}^{-1/2}\mathbf{v}_i = \lambda_{1_i}\left(\tilde{\mathbf{C}}\right)\mathbf{D}^{-1}\mathbf{v}_i \\
&\Longleftrightarrow \quad \mathbf{D}^{-1/2}\tilde{\mathbf{C}}\mathbf{D}^{-1/2}\mathbf{v}_i = k_O^{-1}\lambda_{1_i}\left(\tilde{\mathbf{C}}\right)\mathbf{v}_i \\
&\Longleftrightarrow \quad \mathbf{D}^{-1/2}\tilde{\mathbf{C}}\mathbf{D}^{-1/2}\mathbf{v}_i = \lambda_i\left(\mathbf{D}^{-1/2}\tilde{\mathbf{C}}\mathbf{D}^{-1/2}\right)\mathbf{v}_i
\end{aligned}
$$

where $\tilde{\mathbf{v}}_{1_i}$ is a column of $\tilde{\mathbf{V}}_1$, that is an eigenvector associated with the eigenvalue $\lambda_{1_i}\left(\tilde{\mathbf{C}}\right)$, and is equal to $\tilde{\mathbf{v}}_{1_i} = \begin{bmatrix} \tilde{\mathbf{v}}_{O1_i} \\ \mathbf{0} \end{bmatrix}$, and $\tilde{\mathbf{v}}_{1_i} = \mathbf{D}^{-1/2}\mathbf{v}_i$.
Therefore, it follows that the smallest eigenvalue of $\mathbf{D}^{-1/2}\tilde{\mathbf{C}}\mathbf{D}^{-1/2}$ is proportional to $\lambda_{1_i}\left(\tilde{\mathbf{C}}\right)$, that is $\lambda_{1_{min}}\left(\mathbf{D}^{-1/2}\tilde{\mathbf{C}}\mathbf{D}^{-1/2}\right)$, is equal to the smallest eigenvalue of $\tilde{\mathbf{C}}$, $\lambda_{min}\left(\tilde{\mathbf{C}}\right)$, multiplied by k_O^{-1}, that is $\lambda_{1_{min}}\left(\mathbf{D}^{-1/2}\tilde{\mathbf{C}}\mathbf{D}^{-1/2}\right) = k_O^{-1}\lambda_{min}\left(\tilde{\mathbf{C}}\right)$.

Second, if $\tilde{\mathbf{V}}_{O2} = \mathbf{0}$, it implies that each jth eigenvalue associated with an eigenvector of $\tilde{\mathbf{V}}_2$, that is $\lambda_{2_j}\left(\tilde{\mathbf{C}}\right)$, is proportional by a factor k_S to an eigenvalue of $\mathbf{D}^{-1/2}\tilde{\mathbf{C}}\mathbf{D}^{-1/2}$, that is $\lambda_j\left(\mathbf{D}^{-1/2}\tilde{\mathbf{C}}\mathbf{D}^{-1/2}\right) = k_S^{-1}\lambda_{2_j}\left(\tilde{\mathbf{C}}\right)$, because:

$$
\begin{aligned}
\tilde{\mathbf{C}}\tilde{\mathbf{v}}_{2_j} = \lambda_{2_j}\left(\tilde{\mathbf{C}}\right)\tilde{\mathbf{v}}_{2_j} \quad &\Longleftrightarrow \quad \mathbf{D}^{-1/2}\tilde{\mathbf{C}}\mathbf{D}^{-1/2}\mathbf{v}_j = \lambda_{2_j}\left(\tilde{\mathbf{C}}\right)\mathbf{D}^{-1}\mathbf{v}_j \\
&\Longleftrightarrow \quad \mathbf{D}^{-1/2}\tilde{\mathbf{C}}\mathbf{D}^{-1/2}\mathbf{v}_j = k_S^{-1}\lambda_{2_j}\left(\tilde{\mathbf{C}}\right)\mathbf{v}_j \\
&\Longleftrightarrow \quad \mathbf{D}^{-1/2}\tilde{\mathbf{C}}\mathbf{D}^{-1/2}\mathbf{v}_j = \lambda_j\left(\mathbf{D}^{-1/2}\tilde{\mathbf{C}}\mathbf{D}^{-1/2}\right)\mathbf{v}_j
\end{aligned}
$$

where $\tilde{\mathbf{v}}_{2_j}$ is a column of $\tilde{\mathbf{V}}_2$, that is an eigenvector associated with the eigenvalue $\lambda_{2_j}\left(\tilde{\mathbf{C}}\right)$, and is equal to $\tilde{\mathbf{v}}_{2_j} = \begin{bmatrix} \mathbf{0} \\ \tilde{\mathbf{v}}_{S2_j} \end{bmatrix}$, and $\tilde{\mathbf{v}}_{2_j} = \mathbf{D}^{-1/2}\mathbf{v}_j$.
Therefore, it follows that the smallest eigenvalue of $\mathbf{D}^{-1/2}\tilde{\mathbf{C}}\mathbf{D}^{-1/2}$ proportional to $\lambda_{2_j}\left(\tilde{\mathbf{C}}\right)$, that is $\lambda_{2_{min}}\left(\mathbf{D}^{-1/2}\tilde{\mathbf{C}}\mathbf{D}^{-1/2}\right)$, is equal to the smallest eigenvalue among all the eigenvalues associated with the eigenvectors included in $\tilde{\mathbf{V}}_2$, $\lambda_{2_{min}}\left(\tilde{\mathbf{C}}\right)$, multiplied by k_S^{-1}, that is $\lambda_{2_{min}}\left(\mathbf{D}^{-1/2}\tilde{\mathbf{C}}\mathbf{D}^{-1/2}\right) = k_S^{-1}\lambda_{2_{min}}\left(\tilde{\mathbf{C}}\right)$.

Finally, from the two previous results, that is $\lambda_{1_{min}}\left(\mathbf{D}^{-1/2}\tilde{\mathbf{C}}\mathbf{D}^{-1/2}\right) = k_O^{-1}\lambda_{min}\left(\tilde{\mathbf{C}}\right)$, and $\lambda_{2_{min}}\left(\mathbf{D}^{-1/2}\tilde{\mathbf{C}}\mathbf{D}^{-1/2}\right) = k_S^{-1}\lambda_{2_{min}}\left(\tilde{\mathbf{C}}\right)$, the smallest eigenvalue of the spectrum of $\mathbf{D}^{-1/2}\tilde{\mathbf{C}}\mathbf{D}^{-1/2}$, $\lambda_{min}\left(\mathbf{D}^{-1/2}\tilde{\mathbf{C}}\mathbf{D}^{-1/2}\right)$, is equal to:

$$\begin{aligned}\lambda_{min}\left(\mathbf{D}^{-1/2}\tilde{\mathbf{C}}\mathbf{D}^{-1/2}\right) &= min\left(\lambda_{1_{min}}\left(\mathbf{D}^{-1/2}\tilde{\mathbf{C}}\mathbf{D}^{-1/2}\right), \lambda_{2_{min}}\left(\mathbf{D}^{-1/2}\tilde{\mathbf{C}}\mathbf{D}^{-1/2}\right)\right)\\ &= min\left(k_O^{-1}\lambda_{min}\left(\tilde{\mathbf{C}}\right), k_S^{-1}\lambda_{2_{min}}\left(\tilde{\mathbf{C}}\right)\right)\end{aligned}$$

Therefore, $\lambda_{min}\left(\mathbf{D}^{-1/2}\tilde{\mathbf{C}}\mathbf{D}^{-1/2}\right) = k_O^{-1}\lambda_{min}\left(\tilde{\mathbf{C}}\right)$ if, and only if, $\lambda_{2_{min}}\left(\tilde{\mathbf{C}}\right) \geq \frac{k_S}{k_O}\lambda_{min}\left(\tilde{\mathbf{C}}\right)$, or, in other words, if, and only if, all eigenvalues associated with an eigenvector of $\tilde{\mathbf{V}}_2$ are equal to, or larger than, $\frac{k_S}{k_O}\lambda_{min}\left(\tilde{\mathbf{C}}\right)$.
